# Pyrenosetin D, a New Pentacyclic Decalinoyltetramic Acid Derivative from the Algicolous Fungus *Pyrenochaetopsis* sp. FVE-087

**DOI:** 10.3390/md18060281

**Published:** 2020-05-26

**Authors:** Bicheng Fan, Pradeep Dewapriya, Fengjie Li, Laura Grauso, Martina Blümel, Alfonso Mangoni, Deniz Tasdemir

**Affiliations:** 1 GEOMAR Centre for Marine Biotechnology (GEOMAR-Biotech), Research Unit Marine Natural Products Chemistry, GEOMAR Helmholtz Centre for Ocean Research Kiel, Am Kiel-Kanal 44, 24106 Kiel, Germany; bfan@geomar.de (B.F.); pdewapriya@geomar.de (P.D.); fli@geomar.de (F.L.); mbluemel@geomar.de (M.B.); 2 Dipartimento di Agraria, Università degli Studi di Napoli Federico II, via Università 100, 80055 Portici (NA), Italy; laura.grauso@unina.it; 3 Dipartimento di Farmacia, Università degli Studi di Napoli Federico II, via Domenico Montesano 49, 80131 Napoli, Italy; alfonso.mangoni@unina.it; 4 Faculty of Mathematics and Natural Sciences, Kiel University, Christian-Albrechts-Platz 4, 24118 Kiel, Germany

**Keywords:** *Pyrenochaetopsis*, pyrenosetin, marine fungus, decalinoyltetramic acid, *Fucus vesiculosus*, anticancer

## Abstract

The fungal genus *Pyrenochaetopsis* is commonly found in soil, terrestrial, and marine environments, however, has received little attention as a source of bioactive secondary metabolites so far. In a recent work, we reported the isolation and characterization of three new anticancer decalinoyltetramic acid derivatives, pyrenosetins A–C, from the Baltic *Fucus vesiculosus*-derived endophytic fungus *Pyrenochaetopsis* sp. FVE-001. Herein we report a new pentacyclic decalinoylspirotetramic acid derivative, pyrenosetin D (**1**), along with two known decalin derivatives wakodecalines A (**2**) and B (**3**) from another endophytic strain *Pyrenochaetopsis* FVE-087 isolated from the same seaweed and showed anticancer activity in initial screenings. The chemical structures of the purified compounds were elucidated by comprehensive analysis of HR-ESIMS, FT-IR, [α]_D_, 1D and 2D NMR data coupled with DFT calculations of NMR parameters and optical rotation. Compounds **1–3** were evaluated for their anticancer and toxic potentials against the human malignant melanoma cell line (A-375) and the non-cancerous keratinocyte cell line (HaCaT). Pyrenosetin D (**1**) showed toxicity towards both A-375 and HaCaT cells with IC_50_ values of 77.5 and 39.3 μM, respectively, while **2** and **3** were inactive. This is the third chemical study performed on the fungal genus *Pyrenochaetopsis* and the first report of a pentacyclic decalin ring system from the fungal genus *Pyrenochaetopsis*.

## 1. Introduction

Seaweeds harbor diverse microbial communities, such as bacteria, fungi, bacteriophage, and viruses, forming a complex holobiont [1,2,3]. Endophytic fungi are one of the predominant microbial communities associated with seaweeds and are gaining growing interest as a source of small molecule natural products with high chemical diversity and wide-ranging bioactivity profiles [4]. Over the past decades, several structurally unique metabolites from different classes of natural products, such as polyketides [5], terpenes [6], steroids [7], non-ribosomal peptides [8], and alkaloids [9] were reported from seaweed-derived (algicolous) fungi, with multiple bioactivities, including anticancer [7], antibiotic [9], and antioxidant [10].

Fungal metabolites with a ‘decalin ring system’ represent a large group of architecturally complex structural scaffolds [11]. The decalin ring system is usually substituted with, e.g., a terpene side chain [12] or γ-lactam [13], pyrone [14], diene [15], pyrrolizidine [16], and tetramic acid rings [17]. Decalin derivatives are also intriguing for their remarkable biological activities, including anticancer [18], antiviral [19], and antimicrobial [20]. So far, several decalin derivatives with multiple ring systems (e.g., tricyclic ring system [21]) or rare functional groups (e.g., deoxytetramic acid [17]) have been isolated from different algicolous fungi. As an example, *Ascochyta salicorniae*, an endophytic fungus derived from the green seaweed *Ulva* sp. yielded ascosalipyrrolidinones A and B, unusual decalin derivatives with an ether function on the terminal tetramic acid [17]. Ascosalipyrrolidinone A is a potent antiplasmodial agent that inhibits both the drug-resistant and the drug-sensitive *Plasmodium falciparum* strains K_1_ and NF_54_ (IC_50_ 736 and 378 ng/mL, respectively). It also shows antimicrobial and tyrosine kinase inhibiting activities [17].

As part of our on-going project aiming to investigate the cultivable mycobiome of seaweeds, we have previously isolated 55 epiphytic and endophytic fungi from the Baltic brown alga *Fucus vesiculosus* [22]. In-depth chemical investigation of the endophytic fungus *Pyrenochaetopsis* sp. FVE-001 by using a bioactivity-based molecular networking approach led to the rapid isolation and characterization of three new decalinoylspirotetramic acid derivatives pyrenosetins A–C and the known decalin phomasetin [23]. All four compounds showed notable activity against human malignant melanoma cancer cells (A-375) [23]. In the continuation of our search for new anticancer metabolites from *Fucus vesiculosis*-associated fungi, a second strain of *Pyrenochaetopsis* sp. FVE-087 attracted our attention. In preliminary screenings, the crude extract of this strain also exerted potent anticancer activity [22]. Chemical work-up of this endophyte monitored by anticancer activity against the same melanoma cell line (A-375) resulted in the purification of a new pentacyclic decalinoyltetramic acid derivative, pyrenosetin D (**1**), along with two known decalins wakodecaline A (**2**) and wakodecaline B (**3**). In this study, we report the isolation, detailed structure elucidation, the anticancer activity, and the toxicity of compounds **1**–**3**.

## 2. Results

### 2.1. Strain Identification and Cultivation

The endophytic fungus FVE-087 (GenBank accession number: MH881502) was isolated from the inner thallus of the brown alga *Fucus vesiculosus* collected at Kiel Fjord (Baltic Sea, Germany). The initial taxonomic study of FVE-087 revealed its taxonomical identity at order level, i.e., Pleosporales [22]. The re-amplification of ITS1-5.8S rRNA gene-ITS2 region yielded a longer 408 bp fragment, which enabled its identification at the genus level. A phylogenetic tree was constructed using the nucleotide sequences for related strains (obtained from NCBI database) including the previously identified *Pyrenochaetopsis* sp. FVE-001 from the same seaweed, indicating that the fungus FVE-087 was a close relative of the co-existing fungus *Pyrenochaetopsis* sp. FVE-001. Based on the phylogenetic tree analysis (Figure S1), the strain FVE-087 was confirmed to be a *Pyrenochaetopsis* sp. The fungus FVE-087 was cultivated in the same manner as described in our previous study [23], i.e., in liquid potato dextrose medium (PDM) at 22 °C for 14 days under continuous shaking.

### 2.2. Extraction, Bioactivity Testing, and Isolation

**Figure 1:**
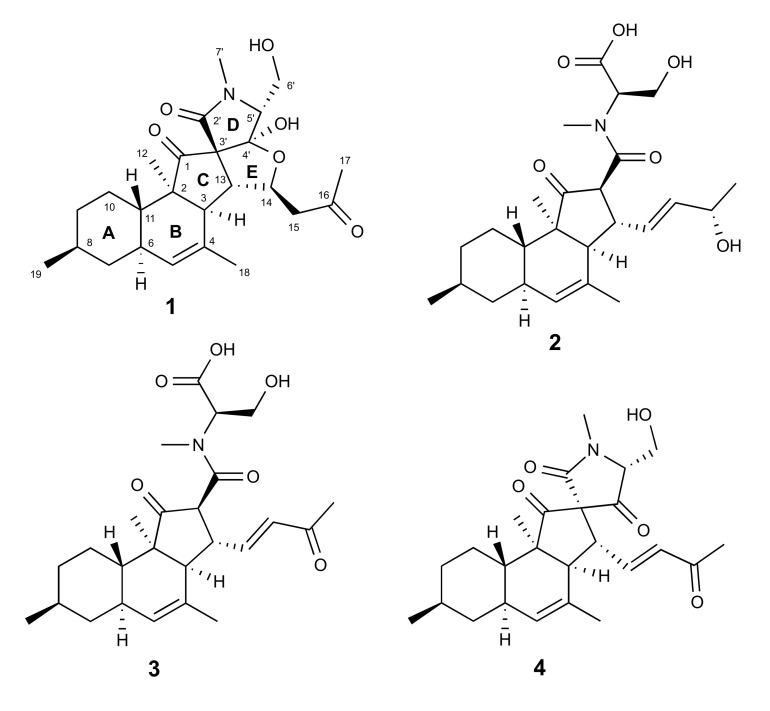
Chemical structures of compounds **1**–**4**.

The culture broth (24 L) was extracted with EtOAc. The crude EtOAc extract of the fungus was subjected to a modified Kupchan partitioning scheme to yield *n*-hexane (KH), CHCl_3_ (KC), and aqueous MeOH (KM) subextracts. All three subextracts were tested for their activity against five cancer cell lines; malignant melanoma (A-375), lung carcinoma (A-549), colorectal adenocarcinoma (HT-29), colorectal carcinoma (HCT-116), and breast cancer cell line (MDA-MB-231). The KC subextract showed the highest inhibitory activity against all tested cell lines (>84% cell growth inhibition at 100 μg/mL) and was selected for a detailed chemical work-up. The KC subextract was fractionated on a C18 SPE cartridge using a 10% gradient of MeOH in water. This yielded 11 subfractions, and the anticancer activity was tracked to late fractions 7–9 (Table S6). Reversed-phase HPLC purification of the fractions 7 and 8, monitored by the anticancer activity against malignant melanoma (A-375) cells, afforded compounds **1**–**3** (Figure 1).

### 2.3. Structure Elucidation

Compound **1** was obtained as a colorless oil. The molecular formula C_25_H_35_NO_6_ that required nine degrees of unsaturation (DoU) was assigned based on the HR-ESIMS spectrum of **1** (Figure S10). The FT-IR spectrum (Figure S11) indicated the presence of hydroxyl (*v*_max_ 3226–3560 cm^-1^), carbonyl (*v*_max_ 1679 and 1734 cm^-1^) and aliphatic ether (*v*_max_ 1064 and 1159 cm^-1^) functions. Comprehensive analysis of the ^1^H NMR data in conjunction with the DEPT-HSQC spectrum (CD_3_CN, Table 1, Figures S4 and S6) revealed the presence of five methyl groups, including one secondary (H_3_-19, δ_H_ 0.89, d, *J* = 6.6 Hz), one tertiary ( H_3_-12, δ_H_ 0.88, s), one olefinic (H_3_-18, δ_H_ 1.76, br s), one methylketone (H_3_-17, δ_H_ 2.09, s), plus an *N*-methyl (H_3_-7′, δ_H_ 2.86, s) signal. In addition, also observed were five pairs of diastereotopic methylene protons, namely H_2_-7 (δ_H_ 0.75, q, *J* = 11.9 Hz and 1.82, m), H_2_-9 (δ_H_ 0.83, dq, *J* = 2.7, 12.6 Hz and 1.71, m), H_2_-10 (δ_H_ 1.02, m and 1.29, dq, *J* = 12.9, 3.0 Hz), H_2_-15 (δ_H_ 2.52, dd, *J* = 16.2, 9.4 Hz and δ_H_ 2.70, dd, *J* = 16.2, 3.1 Hz), and the oxymethylene protons of H_2_-6′ (δ_H_ 3.79, dd, *J* = 11.6, 6.4 Hz and δ_H_ 3.86, dd, *J* = 11.6, 4.1 Hz). The ^1^H NMR spectrum also comprised signals belonging to eight methine protons; this included six aliphatic methine protons which were assigned to H-3 (δ_H_ 2.32, d, *J* = 10.0 Hz), H-6 (δ_H_ 1.84, m), H-8 (1.44, m), H-11 (1.48, ddd, *J* = 11.8, 10.5, 2.7 Hz), H-13 (δ_H_ 2.67, dd, *J* = 10.1, 3.4 Hz), H-5′ (δ_H_ 3.44, dd, *J* = 7.0, 4.1 Hz), plus one olefinic methine proton that appeared as a broad singlet at δ_H_ 5.25 (H-5) and an oxymethine proton that resonated at δ_H_ 4.90 (H-14, dt, *J* = 9.3, 3.3 Hz) (Table 1). The ^13^C NMR spectrum of **1** (Table 1, Figure S5) contained 25 signals accounting for three carbonyls δ_C_ 170.2 (C-2′), 206.8 (C-16), and 213.2 (C-1), and two olefinic carbons at δ_C_ 128.0 (C-5), and 133.2 (C-4), accounting for four DoU. This indicated **1** to be a pentacyclic compound. Comparison of the NMR data of **1** with those of tetracyclic decalinoylspirotetramic acid derivatives pyrenosetins A–C [23] indicated close similarities. Thus, **1** was identified as a pyrenosetin-type decalinoylspirotetramic acid with an additional ring system.

**Figure 2:**
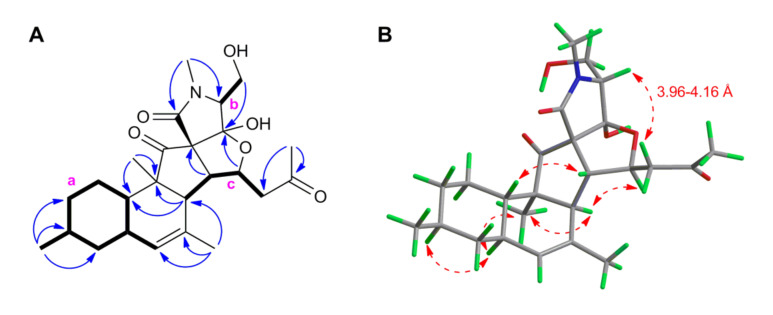
(**A**) Key COSY (**bold**) and HMBC (**blue**) correlations within **1**. **a**–**c**: Spin systems extracted from the COSY spectrum. (**B**) NOESY (**red**) correlations on the lowest-energy DFT conformer of **1**.

In order to identify the position of the fifth ring system and the complete planar structure of **1**, we undertook DQF-COSY and HMBC experiments. The ^1^H-^1^H COSY spectrum of **1** comprised three spin systems (**a**–**c**) (Figure 2A and Figure S7). The largest spin system (**a**) started with the olefinic proton H-5 that coupled with H-6, then included the methyl substituted cyclohexane moiety that terminated with the H-11. The latter proton was in turn coupled with H-6, while H_3_-19 coupled with H-8, completing the structure of the ring **A** with a secondary methyl group attached at C-8. Further detected in the COSY spectrum was a short spin system (**b**) that corresponded to a hydroxyethyl group (H-5′ to H-6′). The final proton network (**c**) covered the protons of H-3, H-13, H-14, and H_2_-15 (Figure 2A and Figure S7).

In-depth analysis of the ^1^H-^13^C HMBC data (Figure 2A and Figure S8) enabled to assemble the three COSY spin systems with quaternary carbons and heteroatoms, hence completing the full structure of **1**. Firstly, the position of the H_3_-19 at C-8 (ring **A**) was further confirmed by the HMBC correlations between H_3_-19 with C-7, C-8, and C-9. The ring **B** of the unsaturated decalin moiety was constructed on the basis of the HMBC cross-peaks observed from H-3 to C-2, C-4, C-5, and C-11, from H-5 to C-3 and C-11, and from the olefinic methyl group H_3_-18 to C-3, C-4, and C-5. The tertiary methyl H_3_-12 was assigned to C-2 based on the key HMBC correlations from H_3_-12 to C-2, C-3, and C-11. Additional HMBC correlations between H_3_-12/C-1, H-3/C-3′, and H-13/C-3′ confirmed the spiro (cyclopentanone) ring system **C** (Figure 2A). The ring **D** comprising of an *N*-methyl tetramic acid was built with the aid of the HMBC correlations between H-5′/C-2′, H-5′/C-4′, H-6′/C-4′, H-6′/C-5′, H_3_-7′/C-2′, H_3_-7′/C-5′. The upfield chemical shift of C-4′ (δ_C_ 110.3) in comparison to other pyrenosetins, such as pyrenosetin C (**4**) suggested the presence in **1** of an acetal or hemiacetal rather than a ketone function. The connection of the ring **D** at C-3′ of the ring **C** was evident due to further HMBC cross-peak seen between H-5′/C-3′.

Pyrenosetins A–C bear a C-16 oxygenated butyl substitution with a double bond Δ^14(15)^ in the side chain (attached at C-13) [23]. The comparison of the ^1^H and ^13^C NMR data of **1** from C-13 to C-15 (Table 1) with those of pyrenosetin C (**4**) [23] clearly showed that **1** lacks the double bond at C-14. Instead, the H-14 was converted to an oxymethine signal (δ_H_ 4.90) that showed a diagnostic HMBC coupling with C-4′ (Figure 2A and Figure S8). This fact, in addition to above mentioned COSY correlations between H-13/H-14 and H-14/H_2_-15 confirmed the fifth tetrahydrofuran ring (**E**). Final HMBC correlations from H_3_-17 to C-15 (δ_C_ 50.3) and C-16 (δ_C_ 206.8) completed the side chain that ended with a methylketone. Thus, the pentacyclic 2D structure of **1** was established as shown in Figure 1.

The relative configuration of the stereogenic centers within **1** was deduced based on NOESY correlations and coupling constant analysis (Figure 2B and Figure S9). The two large axial-axial couplings experienced by H-11 (ddd, *J* = 11.8, 10.5, and 2.7 Hz) established the *trans* junction between rings **A** and **B**. The observed NOESY correlations between H-3/H_3_-12, H-3/H-14, H-6/H-8, H-6/H_3_-12, and H-11/H-13 indicated the α-orientation of H-3, H-6, H-8, H_3_-12, H-14, and the β-orientation of H-11, H-13, and the H_3_-19 methyl group. This left three stereogenic centers (C-3′, C-4′, and C-5′) in the tetramic acid moiety to be assigned.

Assignment of configuration at C-3‘ and C-4′ was based on the high geometric strain of *trans*-5,5-fused ring systems, which are known to be less stable than their *cis* counterparts by more than 6 kcal/mol [24]. As the hemiacetal function at C-4′ would readily open in case of steric strain, the possibility of strained *trans* junctions between rings **C** and **E** and/or between rings **E** and **D** was ruled out, and the requirement of *cis* junctions between rings **C**, **E**, and **D** defined configurations at C-3′ and C-4′ as depicted in structure **1**. The configuration at C-5′ was suggested by a very weak NOE correlation between H-5′ and H-15b (δ_H_ 2.52) (Figure 2B).

As the determined configuration at C-3′ was opposite to that found in the other pyrenosetin analogues (including compound **4**), and configuration at C-5′ was only based on a single weak NOE correlation, the structure of **1** was validated using DFT prediction of NMR parameters. Compound **1**, its epimer at C-3′, namely 3′-*epi*-**1** (which has also opposite configuration at C-4′, to keep the *cis* junction between rings **D** and **E**), and its epimer at C-5′, namely 5′-*epi*-**1** were considered for calculations (see Supplementary Figure S2 for structures, and Materials and Methods Section for details on computational methods). After a molecular dynamics-based conformational search, conformers were optimized at the B3LYP/TZVP/SMD level; then, ^1^H and ^13^C isotropic shieldings were calculated, respectively, at the WP04/aug-cc-pVDZ/PCM and mPW1PW91/6-311+G(2d,p) levels of theory, and scaled to chemical shifts using the linear regression method [25]. Calculations on 3′-*epi*-**1** were soon stopped, because optimized conformers showed energies constantly higher than **1** by more than 10 kcal/mol (as expected, because of the strained *trans* junction between rings **C** and **E**) and root-mean-square deviations (RMSD) of ^13^C NMR chemical shifts of rings **C**, **D**, and **E** constantly over 6 ppm. In contrast, chemical shifts calculated for compound **1** were in excellent agreement with the experimental values (RMSD of 1.66 ppm for ^13^C and 0.076 for ^1^H). While this results provided solid support to the overall structure of **1** and to configurations at C-3′ and C-4′, they did not support the assignment of the configuration at C-5′, because chemical shifts of 5′-*epi*-**1** also showed a comparable agreement with the experiment (RMSD of 1.69 ppm for ^13^C and 0.077 ppm for ^1^H) (Figure S3). Even restricting the comparison to atoms of ring D and/or using DP4+ analysis [26], no clear-cut answer about configuration at C-5′ could be obtained from the predicted chemical shifts. Therefore, coupling constants were examined.

Analysis of structures **1** and 5′-*epi*-**1** showed that the main difference between the two compounds was expected in the ^1^H-^13^C coupling constants of H-5′. In particular, the coupling constant between H-5′ and C-2′ was calculated as 4.3 Hz for **1** and 0.6 Hz for 5′-*epi*-**1** (Table S5). When not measured directly, ^1^H-^13^C coupling constants can be estimated from the intensity of the corresponding HMBC peaks, an intense peak indicating a relatively large *J*_CH_ [27]. The prominent correlation peak observed between H-5′ and C-2′ was not consistent with the small 0.6 Hz coupling constant predicted for 5′-*epi*-**1,** and conclusively determined the relative stereochemistry of pyrenosetin D as in structure **1**. Finally, the observed weak NOE correlation between H-5 and H-15b was in good agreement with the distance between 3.96 and 4.16 Å of these protons measured for the DFT optimized conformers of **1** (Figure 2B).

The absolute configuration of compound **1** was determined on the basis of its predicted optical rotation (OR). DFT prediction of OR is a valuable, although not as general, alternative to ECD for determining the absolute configuration of natural products, and can be reliably used for this purpose provided that (i) the magnitude of the measured OR is not close to zero and (ii) the sign of the calculated OR is the same for all, or at least for most conformers [28]. The OR of compound **1** was predicted at the B3LYP/TZVP/PCM(MeOH) level of theory; the calculated [α]_D_ (as the Boltzmann average of individual conformers) was -104, compared to the experimental value -56. In addition, the OR values calculated for individual conformers were all negative, and with similar magnitude. These results strongly supported the absolute configuration of pyrenosetin D as shown in Figure 1.

The known compounds **2** and **3** were identified as wakodecalines A and B (Figure 1), respectively, based on their HR-ESIMS and MS/MS data, plus by comparison of their 1D/2D NMR and [α]_D_ data with those reported in the literature [29].

### 2.4. Bioactivity Tests

Due to the limited availability, compounds **1**–**3** were tested for their inhibitory activity against only one cancer cell line, i.e., the human malignant melanoma cell line (A-375). The general toxicity of the isolated metabolites was assessed against the human keratinocyte cells (HaCaT). Compound **1** exhibited moderate anticancer activity against the A-375 cells (IC_50_ value 77.5 μM), but it was also toxic towards the HaCaT cells (IC_50_ value of 39.3 μM). The known compounds **2** and **3** did not exert anticancer or toxic effects, even at the highest test concentration of 200 μM.

## 3. Discussion

*Pyrenochaetopsis* is a ubiquitous fungal genus found in both terrestrial and marine environments [30,31]. However, the members of this genus have remained almost fully unexplored for their bioactive secondary metabolites. To our knowledge, the very first chemical study was published in 2017 by Nogawa et al. on a *Pyrenochaetopsis* sp. isolated from a soil sample collected in Japan [29]. This work reported wakodecalines A, B, two new tricyclic decalin derivatives with a spiro pentanone ring (C) and *N*-methylated terminal serine moiety as well as phomasetin, a known decalin compound with a terminal tetramic acid function [29]. The lack of any other investigation in the literature on chemical constituents or biological activity has initiated our interest into this unexplored fungal taxon. In a very recent study, we demonstrated that, when cultured in liquid potato dextrose medium, the algicolous *Pyrenochaetopsis* sp. FVE-001 produces pyrenosetins A–C, new tetracyclic decalin derivatives with good activity against malignant melanoma cells (A-375) [23]. In addition, also isolated and identified from this fungus was the known compound phomasetin [23].

The ‘decalin’ moiety is a common structural motif in an array of marine fungal natural products [13,32,33]. It has been proposed that the decalin ring serves as a primary ring system or a scaffold to form polycyclic metabolites, with bicyclic decalins being the most common [11,13,32,33,34,35]. The chemical investigation of the marine sponge-derived fungus *Trichoderma harzianum* in 1993 led to the isolation of the first bicyclic decalin derivative trichoharzin isolated from a marine fungus. Trichoharzin contains rare alkyl and acyl moieties [36]. So far, more than 50 decalin derivatives have been reported from marine fungi [11,13,32,33,34,35,36,37]. The marine-derived bicyclic decalin derivatives often incorporate additional ring systems, e.g., pyrone [37], cyclopentanone [13], and tetramic acid [34] to lead tricyclic [35,37], tetracyclic [13], and pentacyclic scaffolds [34]. Of all, tetramic acid represents one of the most common substituents [13,34,38]. Pyrenosetins A–C, which we recently reported from *Pyrenochaetopsis* sp. FVE-001, an endophyte of the Baltic brown alga *Fucus vesiculosus*, represent one of the most complex tetracyclic decalinoylspirotetramic acids reported to date [23]. Such complex decalin scaffold is rare in fungi. To our knowledge, only a few fungal genera such as *Fusarium* sp. [34,39], *Alternaria* sp. [13], *Diaporthe* sp. [40], and *Pyrenochaetopsis* sp. [23] have been reported to produce such type of unique molecules. Decalinoyltetramic acid derivatives are also intriguing for remarkable biological activities they exhibit. For example, altercrasins D and E isolated from sea-urchin-derived *Alternaria* sp., have shown potent activity against murine P388 leukemia, human HL-60 leukemia, and murine L1210 leukemia cell lines [13]. However, most of the decalin derivatives suffer from poor selectivity, i.e., they also possess toxicity towards non-cancerous cells. This is a common characteristic of mycotoxins [34,41].

Our previous research on pyrenosetins highlighted the importance of the side chain attached at C-13 for their bioactivity [23]; both the anticancer activity and the general toxicity were significantly reduced with the oxidation of the C-16 hydroxyl group to a ketone. Notably, wakodecalines (**2** and **3**) that lack the terminal tetramic acid moiety did not show any bioactivity against A-375 cells, even at the highest test concentrations. This suggests that both side chain and the tetramic acid moiety are crucial for bioactivity of the decalin derivatives. Additionally, the presence of the additional tetrahydrofuran ring as found in **1** may be improving the activity against A-375 cells (IC_50_ 77.5 μM) when compared to the anticancer activity of pyrenosetin C on the same cell line (IC_50_ 140.3 μM) [23]. A previous study showed that fusarisetin A that possesses a tetrahydrofuran ring exhibits higher activity against the invasive breast cancer cell line MDA-MB-231 than its precursor equisetin [42].

In conclusion, a new cytotoxic pentacyclic decalinoylspirotetramic acid derivative, pyrenosetin D (**1**) and two known and non-toxic tricyclic metabolites, wakodecalines A (**2**) and B (**3**), were purified from the algicolous fungus *Pyrenochaetopsis* sp. To our knowledge, only fusarisetins that were isolated from *Fusarium* sp. possess somehow similar pentacyclic skeleton [34,39]. This is the third study performed on the chemical constituents of the fungal genus *Pyrenochaetopsis* and the first report of a pentacyclic decalin ring system from *Pyrenochaetopsis* species. Further investigation of the anticancer activity, selectivity, and mechanism of cytotoxic action of our metabolites might expand our knowledge of the structure–activity relationship of decalinoyltetramic acid derivatives.

## 4. Materials and Methods

### 4.1. General Procedures

FT-IR spectra were recorded on a PerkinElmer Spectrum Two FT-IR spectrometer (PerkinElmer, Boston, MA, USA). Specific rotation ([α]_D_) values were measured in MeOH on a Jasco P-2000 polarimeter (Jasco, Pfungstadt, Germany). NMR data were recorded on a Bruker AV 600 spectrometer (600 and 150 MHz for ^1^H and ^13^C NMR, respectively, Bruker®, Billerica, MA, USA). The residual solvent signals were detected in NMR spectra as internal references: *δ*_H_ 1.94/*δ*_C_ 118.3 and *δ*_C_ 1.3 (CD_3_CN). 4-Dimethyl-4-silapentane-1-sulfonic acid (DSS) was used as an internal standard. HRESIMS was recorded on a microTOF II-high-performance TOF-MS system (Bruker®, Billerica, MA, USA) equipped with an electrospray ionization source. Solid-phase extraction (SPE) was performed on a C18 cartridge (50 μm, 65Å, Phenomenex, 411 Madrid Avenue, Torrance, CA, USA). HPLC separations were performed on a VWR Hitachi Chromaster system (VWR International, Allison Park, PA, USA) consisting of a 5430 diode array detector (VWR International, Allison Park, PA, USA), a 5310 column oven, a 5260 autosampler, and a 5110 pump. The eluents used for HPLC separations were milli Q water (A) and MeCN (B). Routine HPLC separations were performed on a semi-preparative C18 monolithic column (Onyx, 100 × 10 mm, Phenomenex, Torrance, CA, USA) and an analytical synergi polar-RP 80 Å LC column (250 × 4.6 mm, Phenomenex, Torrance, CA, USA). The organic solvents used for chemical analysis were of HPLC grade (ITW Reagents, Germany). An in-house Arium^®^ Water Purification Systems (Sartorius, Germany) was used for the preparation of milli Q water. Solvents used in extraction, Kupchan partition, and purification (including EtOAc, *n*-hexane, MeOH and MeCN) were purchased from VWR International GmbH (Hannover, Germany). Potato extract and dextrose that used for fungal cultivation were purchased from Sigma-Aldrich (Schnelldorf, Germany) and Merck (Darmstadt, Germany), respectively. Agar was purchased from Applichem (Darmstadt, Germany).

### 4.2. Strain Identification and Cultivation

The fungal strain *Pyrenochaetopsis* sp. FVE-087 (GenBank accession number: MH881502) was obtained from *Fucus vesiculosus* specimens that were collected in Falckenstein Beach (54°23′22.6″ N, 10°11′26.4″ E), Kiel Fjord, Baltic Sea, in December 2015, Germany [22]. The fungus was identified by morphological observation, analysis of the ITS1-5.8S rRNA gene-ITS2 region and by building a phylogenetic tree with 14 related strains including the *Pyrenochaetopsis* sp. FVE-001, using the method described in our previous work [23]. The initial cultures were maintained on potato dextrose agar plates (PDA: potato extract 4 g, dextrose 20 g, agar 15g for 1 L, pH 5.6). After 3 days of pre-cultivation, pieces of mycelia were cut into small segments and aseptically inoculated into an Erlenmeyer flask (300 mL) that contained 100 mL of potato dextrose broth media (PDM: potato extract 4 g, dextrose 20 g for 1 L; pH 5.6). After 7 days inoculation, 1 mL liquid seed was added into Erlenmeyer flasks (2 L), each containing 800 mL PDM. A 24 L culture broth was fermented at 22 °C for 14 days on a rotary shaker at 120 rpm.

### 4.3. Extraction and Isolation

The culture broth was partitioned against the same volume of EtOAc twice at room temperature. The EtOAc phase was evaporated to dryness under reduced pressure to afford 17.64 g yellow oily extract. The extract was subjected to a modified Kupchan partition scheme to yield three subextracts, *n*-hexane (KH, 6.93 g), CHCl_3_ (KC, 4.57 g), and aqueous MeOH (KM, 440.5 mg). All three subextracts were tested for their bioactivity against five cancer cell lines and non-cancerous cell line HaCaT. The KC subextract showed high anticancer bioactivity against all five cancer cell lines (>84% inhibition rate at 100 μg/mL) and was fractionated on a C18-SPE column eluting with 10% stepwise gradient of MeOH in water (0–100%) to afford 11 fractions (F0–F10). Anticancer bioactivity was tracked to fractions F7–F9 (Table S6). The fraction 7 (F7, 138 mg) was subjected to semi-preparative RP–HPLC equipped with an Onyx monolithic C18 column. Elution with a gradient MeCN:H_2_O mixture (25–35% MeCN over 30 min, flow 3.0 mL/min) yielded eight subfractions (F7-1 to F7-8). The F7-7 (10.8 mg) was further purified by RP-HPLC on an analytical synergi polar-RP 80 Å column eluting with MeCN:H_2_O (52% isocratic MeCN over 19 min, flow 1.0 mL/min) to yield wakodecaline B (**3**, 2.4 mg, t_R_ 8.8 min) and pyrenosetin D (**1**, 1.2 mg, t_R_ 10.3 min). The subfraction 8 (F8, 369.6 mg) was chromatographed by RP-HPLC equipped with Onyx monolithic C18 column using MeCN:H_2_O mixtures (40% isocratic MeCN over 28 min and gradual increase to 60% MeCN by 40 min, flow 3.0 mL/min) to yield 14 subfractions (F8-1 to 14). Wakodecaline A (**2**) was tracked to F8-3 (7 mg). This fraction was subjected to RP-HPLC on an analytical synergi polar-RP 80 Å LC column using an isocratic mixture of MeCN:H_2_O (45:55) (flow 1.0 mL/min) to yield wakodecaline A (**2**, 2.9 mg, t_R_ 9.5 min).

*Pyrenosetin D* (**1**): Colorless oil; [α]^20^_D_ −56 (*c* 0.10, MeOH); IR (oil) *v*_max_ 3226-3560, 2947, 2921, 1734, 1679, 1460, 1406, 1377, 1159, 1064 cm^−1^. ^1^H NMR (CD_3_CN, 600 MHz) and ^13^C NMR (CD_3_CN, 150 MHz) are shown in Table 1; HR-ESIMS found *m/z* 446.2520 [M + H]^+^, C_25_H_36_NO_6_, calculated for 446.2537.

### 4.4. Computational Details

Conformational search for **1** and 5′-*epi*-**1** was based on a 10-ns molecular dynamics (MD) simulation at 600 K as previously described [43]. The MD simulation generated 101 and 102 different conformers for **1** and 5′-*epi*-**1**, respectively, within 5 kcal/mol from the lowest energy conformer. Optimizations of geometries from MD were performed using density functional theory (DFT) with the Gaussian 16 program [44], the B3LYP functional, the 6-31G(d,p) basis set, and the SMD model for the solvent, ACN. This resulted in 15 and 17 conformers for **1** and 5′-*epi*-**1**, respectively, within 3 kcal/mol from the lowest energy conformer. Finally, these conformers were further optimized at the B3LYP/TZVP/SMD level of theory, giving a final set of 10 significantly populated (population > 1% at 298 K) conformers for **1** and 11 significantly populated conformers for 5′-*epi*-**1**. Vibrational frequency analysis revealed no imaginary frequencies, confirming that all conformers were in a true energy minimum. The Cartesian coordinates and relative energies of the conformers are reported in Tables S1 and S2. These conformational ensembles were used for all the subsequent calculations. 

NMR isotropic shieldings of **1** and 5′-*epi*-**1** were calculated using WP04/aug-cc-pVDZ/PCM for ^1^H and mPW1PW91/6-311+G(2d,p) for ^13^C, which have been shown to be most accurate levels of theory, among those tested, for NMR data acquired in CD_3_CN [45]. Average shieldings were obtained by Boltzmann statistics based on internal energies of conformers. Finally, shieldings were scaled to chemical shifts using linear regression [25]. The results are reported in Tables S3 and S4. Coupling constants were calculated at the B3LYP/6-31G(d,p) level and using the keyword "mixed", which augments the basis set for the calculation of the Fermi Contact term, and averaged by Boltzmann statistics. The results are reported in Table S4.

Optical rotations of DFT-optimized conformer of **1** were calculated using TDDFT at the B3LYP/TZVP/PCM(MeOH) level; the results can be found in Table S1. The Boltzmann mean of individual optical rotations gave [α]_D_ = −105.

### 4.5. Biological Assays

The bioactivity tests were performed as described previously [23]. The crude extract, the subextracts and the SPE fractions of the KC subextract were tested in vitro against five human cancer cell lines: colorectal adenocarcinoma cell line HT-29 (DSMZ, Braunschweig, Germany), malignant melanoma cell line A-375 (CLS, Eppelheim, Germany), colon cancer cell line HCT-116 (DSMZ, Braunschweig, Germany), lung carcinoma cell line A-549 (CLS, Eppelheim, Germany), human breast cancer line MDA-MB-231 (CLS, Eppelheim, Germany), as well as for the non-cancerous human keratinocyte line HaCaT (CLS, Eppelheim, Germany) at a concentration of 100 μg/mL. The bioactivity of the extracts was evaluated by monitoring the metabolic activity using the CellTiterBlue Cell Viability Assay (Promega, Mannheim, Germany). HT-29 and HaCaT cells were cultivated in RPMI medium, A-549 and MDA-MB-231 cells in DMEM:Ham’s F12 medium (1:1) supplemented with 15mM HEPES and A-375 and HCT-116 cells in DMEM medium supplemented with 4.5 g/L D-Glucose and 110 mg/L sodium pyruvate. All media were supplemented with L-glutamine, 10% fetal bovine serum, 100 U/mL penicillin, and 100 mg/mL streptomycin. The cultures were maintained at 37 °C under a humidified atmosphere and 5% CO_2_. The cell lines were transferred every 3 or 4 days. For the experimental procedure, the cells were seeded in 96-well plates at a concentration of 10,000 cells per well. A stock solution of 40 mg/mL in DMSO was prepared for each extract. After 24 h incubation, the medium was removed from the cells and 100 μL fresh medium containing the test samples was added. Each sample was prepared in duplicate once. Doxorubicin as a standard therapeutic drug was used as a positive control, 0.5% DMSO and growth media were used as negative controls. Following compound addition, plates were cultured at 37 °C for 24 h. Afterward, the assay was performed according to the manufacturer’s instructions and measured using the microplate reader Tecan Infinite M200 at excitation 560 nm and emission of 590 nm. For determination of IC_50_ values, a dilution series of the extracts were prepared and tested, as described before for the crude extract. The IC_50_ values were calculated by Excel as the concentration that shows 50% inhibition of the viability based on a negative control (no compound) and compared with the positive control (doxorubicin).

## Figures and Tables

**Table 1:** ^1^H NMR (600 MHz) and ^13^C NMR (150 MHz) data of compound **1** (CD_3_CN)_._

No.		1	
		δ_H_, mult (*J* in Hz)	δ_C_
1		-	213.2
2		-	56.4
3		2.32, d (10.0)	54.8
4		-	133.2
5		5.25, br s	128.0
6		1.84, m	37.7
7	eq	1.82, m	42.9
	ax	0.75, q (11.9)	
8		1.44, m	33.5
9	eq	1.71, m	36.0
	ax	0.83, dq (2.7, 12.6)	
10	eq	1.29, dq (12.9, 3.0)	25.8
	ax	1.02, m	
11		1.48, ddd (11.8, 10.5, 2.7)	37.9
12		0.88, s	14.2
13		2.67, dd (10.1, 3.4)	57.0
14		4.90, dt (9.3, 3.3)	83.7
15	a	2.70, dd (16.2, 3.1)	50.3
	b	2.52, dd (16.2, 9.4)	
16		-	206.8
17		2.09, s	30.8
18		1.76, br s	23.2
19		0.89, d (6.6)	22.5
2′		-	170.2
3′		-	75.3
4′		-	110.3
5′		3.44, dd (7.0, 4.1)	68.3
6′	a	3.86, dd (11.6, 4.1)	60.4
	b	3.79, dd (11.6, 6.4)	
7′		2.86, s	29.5

## Supplementary Materials

The following are available online at https://www.mdpi.com/1660-3397/18/6/281/s1; NMR, HRESIMS, and FT-IR spectra of compound **1**. Tables with detailed information of computational results.

## References

[1] Flewelling A.J., Johnson J.A., Gray C.A. (2013). Isolation and bioassay screening of fungal endophytes from North Atlantic marine macroalgae. Bot. Mar..

[2] Zuccaro A., Schoch C.L., Spatafora J.W., Kohlmeyer J., Draeger S., Mitchell J.I. (2008). Detection and identification of fungi intimately associated with the brown seaweed *Fucus serratus*. Appl. Environ. Microbiol..

[3] Egan S., Harder T., Burke C., Steinberg P., Kjelleberg S., Thomas T. (2013). The seaweed holobiont: Understanding seaweed–bacteria interactions. FEMS Microbiol. Rev..

[4] Zhang P., Li X., Wang B.-G. (2016). Secondary metabolites from the marine algal-derived endophytic fungi: Chemical diversity and biological activity. Planta Med..

[5] Son B.W., Choi J.S., Kim J.C., Nam K.W., Kim D.-S., Chung H.Y., Kang J.S., Choi H.D. (2002). Parasitenone, a new epoxycyclohexenone related to gabosine from the marine-derived fungus *Aspergillus parasiticus*. J. Nat. Prod..

[6] Almeida C., Elsaedi S., Kehraus S., König G.M. (2010). Novel bisabolane sesquiterpenes from the marine-derived fungus *Verticillium tenerum*. Nat. Prod. Commun..

[7] Cui C.-M., Li X.-M., Meng L., Li C.-S., Huang C.-G., Wang B.-G. (2010). 7-Nor-ergosterolide, a pentalactone-containing norsteroid and related steroids from the marine-derived endophytic *Aspergillus ochraceus* EN-31. J. Nat. Prod..

[8] Komatsu K., Shigemori H., Kobayashi J. (2001). Dictyonamides A and B, new peptides from marine-derived fungus. J. Org. Chem..

[9] Du F.-Y., Li X.-M., Li C.-S., Shang Z., Wang B.-G. (2012). Cristatumins A–D, new indole alkaloids from the marine-derived endophytic fungus *Eurotium cristatum* EN-220. Bioorg. Med. Chem. Lett..

[10] Abdel-Lateff A., Klemke C., König G.M., Wright A.D. (2003). Two new xanthone derivatives from the algicolous marine fungus *Wardomyces anomalus*. J. Nat. Prod..

[11] Li G., Kusari S., Spiteller M. (2014). Natural products containing ‘decalin’ motif in microorganisms. Nat. Prod. Rep..

[12] Sobolevskaya M.P., Leshchenko E.V., Hoai T.P.T., Denisenko V.A., Dyshlovoy S.A., Kirichuk N.N., Khudyakova Y.V., Kim N.Y., Berdyshev D.V., Pislyagin E.A. (2016). Pallidopenillines: Polyketides from the alga-derived fungus *Penicillium thomii* Maire KMM 4675. J. Nat. Prod..

[13] Yamada T., Tanaka A., Nehira T., Nishii T., Kikuchi T. (2019). Altercrasins A–E, decalin derivatives, from a sea-urchin-derived *Alternaria* sp.: Isolation and structural analysis including stereochemistry. Mar. Drugs.

[14] Jenkins K.M., Toske S.G., Jensen P.R., Fenical W. (1998). Solanapyrones E-G, antialgal metabolites produced by a marine fungus. Phytochemistry.

[15] Nguyen H.P., Zhang D., Lee U., Kang J.S., Choi H.D., Son B.W. (2007). Dehydroxychlorofusarielin B, an antibacterial polyoxygenated decalin derivative from the marine-derived fungus *Aspergillus* sp.. J. Nat. Prod..

[16] Nogawa T., Kawatani M., Uramoto M., Okano A., Aono H., Futamura Y., Koshino H., Takahashi S., Osada H. (2013). Pyrrolizilactone, a new pyrrolizidinone metabolite produced by a fungus. J. Antibiot..

[17] Osterhage C., Kaminsky R., König G.M., Wright A.D. (2000). Ascosalipyrrolidinone A, an antimicrobial alkaloid, from the obligate marine fungus *Ascochyta salicorniae*. J. Org. Chem..

[18] Yamada T., Mizutani Y., Umebayashi Y., Inno N., Kawashima M., Kikuchi T., Tanaka R. (2014). Tandyukisin, a novel ketoaldehyde decalin derivative, produced by a marine sponge-derived *Trichoderma harzianum*. Tetrahedron Lett..

[19] Singh S.B., Zink D.L., Goetz M.A., Dombrowski A.W., Polishook J.D., Hazuda D.J. (1998). Equisetin and a novel opposite stereochemical homolog phomasetin, two fungal metabolites as inhibitors of HIV-1 integrase. Tetrahedron Lett..

[20] Alfatafta A.A., Gloer J.B., Scott J.A., Malloch D. (1994). Apiosporamide, a new antifungal agent from the coprophilous fungus *Apiospora montagnei*. J. Nat. Prod..

[21] Duong T.-H., Nguyen H.-H., Le T.-T., Tran T.-N., Sichaem J., Nguyen T.-T., Nguyen T.-P., Mai D.-T., Nguyen H.-H., Le H.-D. (2020). Subnudatones A and B, new *trans*-decalin polyketides from the cultured lichen mycobionts of *Pseudopyrenula subnudata*. Fitoterapia.

[22] Fan B., Parrot D., Blümel M., Labes A., Tasdemir D. (2019). Influence of OSMAC-based cultivation in metabolome and anticancer activity of fungi associated with the brown alga *Fucus vesiculosus*. Mar. Drugs.

[23] Fan B., Dewapriya P., Li F., Blümel M., Tasdemir D. (2020). Pyrenosetins A–C, new decalinoylspirotetramic acid derivatives isolated by bioactivity-based molecular networking from the seaweed-derived fungus *Pyrenochaetopsis* sp. FVE-001. Mar. Drugs.

[24] Carey F.A., Sundberg R.J. (1990). Advanced Organic Chemistry.

[25] Moosmann P., Ueoka R., Grauso L., Mangoni A., Morinaka B.I., Gugger M., Piel J. (2017). Cyanobacterial *ent*-sterol-like natural products from a deviated ubiquinone pathway. Angew. Chem. Int. Ed. Engl..

[26] Grimblat N., Zanardi M.M., Sarotti A.M. (2015). Beyond DP4: An improved probability for the stereochemical assignment of isomeric compounds using quantum chemical calculations of NMR shifts. J. Org. Chem..

[27] Ciminiello P., Dell′Aversano C., Dello Iacovo E., Fattorusso E., Forino M., Grauso L., Tartaglione L. (2012). Stereochemical studies on ovatoxin-a. Chem. Eur. J..

[28] Grauso L., Teta R., Esposito G., Menna M., Mangoni A. (2019). Computational prediction of chiroptical properties in structure elucidation of natural products. Nat. Prod. Rep..

[29] Nogawa T., Kato N., Shimizu T., Okano A., Futamura Y., Takahashi S., Osada H. (2017). Wakodecalines A and B, new decaline metabolites isolated from a fungus *Pyrenochaetopsis* sp. RK10-F058. J. Antibiot..

[30] De Gruyter J., Woudenberg J.H.C., Aveskamp M.M., Verkley G.J.M., Groenewald J.Z., Crous P.W. (2013). Redisposition of *Phoma*-like anamorphs in Pleosporales. Stud. Mycol..

[31] De Gruyter J., Woudenberg J.H.C., Aveskamp M.M., Verkley G.J.M., Groenewald J.Z., Crous P.W. (2010). Systematic reappraisal of species in *Phoma* section *Paraphoma*, *Pyrenochaeta* and *Pleurophoma*. Mycologia.

[32] Klemke C., Kehraus S., Wright A.D., König G.M. (2004). New secondary metabolites from the marine endophytic fungus *Apiospora montagnei*. J. Nat. Prod..

[33] Wu B., Wiese J., Labes A., Kramer A., Schmaljohann R., Imhoff J.F. (2015). Lindgomycin, an unusual antibiotic polyketide from a marine fungus of the Lindgomycetaceae. Mar. Drugs.

[34] Zhao D., Han X., Wang D., Liu M., Gou J., Peng Y., Liu J., Li Y., Cao F., Zhang C. (2019). Bioactive 3-decalinoyltetramic acids derivatives from a marine-derived strain of the fungus *Fusarium equiseti* D39. Front. Microbiol..

[35] Afiyatullov S.S., Leshchenko E.V., Berdyshev D.V., Sobolevskaya M.P., Antonov A.S., Denisenko V.A., Popov R.S., Pivkin M.V., Udovenko A.A., Pislyagin E.A. (2017). Zosteropenillines: Polyketides from the marine-derived fungus *Penicillium thomii*. Mar. Drugs.

[36] Kobayashi M., Uehara H., Matsunami K., Aoki S., Kitagawa I. (1993). Trichoharzin, a new polyketide produced by the imperfect fungus *Trichoderma harzianum* separated from the marine sponge *Micale cecilia*. Tetrahedron Lett..

[37] Ma Y., Li J., Huang M., Liu L., Wang J., Lin Y. (2015). Six new polyketide decalin compounds from mangrove endophytic fungus *Penicillium aurantiogriseum* 328#. Mar. Drugs.

[38] Yamada T., Kikuchi T., Tanaka R. (2015). Altercrasin A, a novel decalin derivative with spirotetramic acid, produced by a sea urchin-derived *Alternaria* sp.. Tetrahedron Lett..

[39] Jang J.-H., Asami Y., Jang J.-P., Kim S.-O., Moon D.O., Shin K.-S., Hashizume D., Muroi M., Saito T., Oh H. (2011). Fusarisetin A, an acinar morphogenesis inhibitor from a soil fungus, *Fusarium* sp. FN080326. J. Am. Chem. Soc..

[40] Pornpakakul S., Roengsumran S., Deechangvipart S., Petsom A., Muangsin N., Ngamrojnavanich N., Sriubolmas N., Chaichit N., Ohta T. (2007). Diaporthichalasin, a novel CYP3A4 inhibitor from an endophytic *Diaporthe* sp.. Tetrahedron Lett..

[41] Pitt J.I., Miller J.D. (2017). A concise history of mycotoxin research. J. Agric. Food Chem..

[42] Xu J., Caro-Diaz E.J.E., Lacoske M.H., Hung C.-I., Jamora C., Theodorakis E.A. (2012). Fusarisetin A: Scalable total synthesis and related studies. Chem. Sci..

[43] Grauso L., Li Y., Scarpato S., Shulha O., Rárová L., Strnad M., Teta R., Mangoni A., Zidorn C. (2020). Structure and conformation of zosteraphenols, tetracyclic diarylheptanoids from the seagrass *Zostera marina*: An NMR and DFT Study. Org. Lett..

[44] Frisch M.J., Trucks G.W., Schlegel H.B., Scuseria G.E., Robb M.A., Cheeseman J.R., Scalmani G., Barone V., Petersson G.A., Nakatsuji H. (2019). Gaussian 16. Revision C.01..

[45] Pierens G.K. (2014). ^1^H and ^13^C NMR scaling factors for the calculation of chemical shifts in commonly used solvents using density functional theory. J. Comput. Chem..

