# Supplementary material for: Pyrenosetin D, a New Pentacyclic Decalinoyltetramic Acid Derivative from the Algicolous Fungus Pyrenochaetopsis sp. FVE-087
Source: Mar Drugs. 2020 May 26;18(6):281. doi: 10.3390/md18060281 (PMC7344976; doi:10.3390/md18060281)
Supplement: Supplementary file 1 [file marinedrugs-18-00281-s001.pdf]

Supporting Information

Pyrenosetin D, a New Pentacyclic Decalinoyltetramic Acid Derivative from the Algicolous Fungus *Pyrenochaetopsis* sp. FVE-087

Bicheng Fan [1], Pradeep Dewapriya [1], Fengjie Li [1], Laura Grauso [2], Martina Blümel [1], Alfonso Mangoni [3], and Deniz Tasdemir [1, 4*]

[1] GEOMAR Centre for Marine Biotechnology (GEOMAR-Biotech), Research Unit Marine Natural Products Chemistry, GEOMAR Helmholtz Centre for Ocean Research Kiel, Am Kiel-Kanal 44, 24106 Kiel, Germany; bfan@geomar.de (B.F.); pdewapriya@geomar.de (P.D.); fli@geomar.de (F. L.); mbluemel@geomar.de (M.B.)

[2] Dipartimento di Agraria, Università degli Studi di Napoli Federico II, via Università 100, 80055 Portici (NA), Italy; laura.grauso@unina.it (L.G.)

[3] Dipartimento di Farmacia, Università degli Studi di Napoli Federico II, via Domenico Montesano 49, 80131 Napoli, Italy; alfonso.mangoni@unina.it (A.M.)

[4] Faculty of Mathematics and Natural Sciences, Kiel University, Christian-Albrechts-Platz 4, 24118 Kiel, Germany

* Correspondence: dtasdemir@geomar.de; Tel.: +49-431-600-4430

List of Figures:

Figure S1. Molecular phylogenetic analysis of *Pyrenochaetopsis sp.* FVE-087 by maximum likelihood method

Figure S2. Structures of compounds **1**, 3'-*epi*-**1**, and 5'-*epi*-**1**

Figure S3. Error of scaled predicted chemical shifts compared to experimental chemical shifts

Figure S4. ^{1}H NMR spectrum of compound **1** (600 MHz, CD_3CN)

Figure S5. ^{13}C NMR spectrum of compound **1** (150 MHz, CD_3CN)

Figure S6. DEPT-HSQC spectrum of compound **1** (600 MHz, CD_3CN)

Figure S7. COSY spectrum of compound **1** (600 MHz, CD_3CN)

Figure S8. HMBC spectrum of compound **1** (600 MHz, CD_3CN)

Figure S9. NOESY spectrum of compound **1** (600 MHz, CD_3CN)

Figure S10. HR-ESIMS spectrum of compound **1**

Figure S11. FT-IR spectrum of compound **1**

List of Tables:

Table S1. Cartesian coordinates, energies, population at 298 K, and calculated optical rotations of significantly populated conformers of compond **1**. Conformers were optimized at the B3LYP/TZVP/SMD(ACN) level; optical rotation were calculated at the B3LYP/TZVP/SMD(MeOH) level

Table S2. Cartesian coordinates, energies, and population at 298 K of significantly populated conformers of compound 5'-*epi*-**1**. Conformers were optimized at the B3LYP/TZVP/SMD(ACN) level

Table S3. Isotropic shieldings of significantly populated conformers of compound **1**, average isotropic shieldings over conformers, linearly scaled calculated chemical shifts, experimental chemical shifts, and errors

Table S4. Isotropic shieldings of significantly populated conformers of compound 5'-*epi*-**1**, average isotropic shieldings over conformers, linearly scaled calculated chemical shifts, experimental chemical shifts, and errors

Table S5. Selected coupling constants of compounds **1** and 5'-*epi*-**1** calculated at the B3LYP/6-31G(d,p) level and relative intensities of the corresponding HMPC peaks

Table S6. Bioactivity of Kupchan subextracts (KM, KC, KH) and C18 SPE fractions (F0-F10) obtained from *Pyrenochaetopsis* FVE-087 against cancer cell lines (A-375, A-549, HT-29, HCT-116, MB-231) and HaCaT cells. The activity is expressed as % inhibition at 100 μg/mL test concentration. Reference compound: Doxorubicin.

Figure S1. Molecular phylogenetic analysis of *Pyrenochaetopsis sp.* FVE-087 by maximum likelihood method

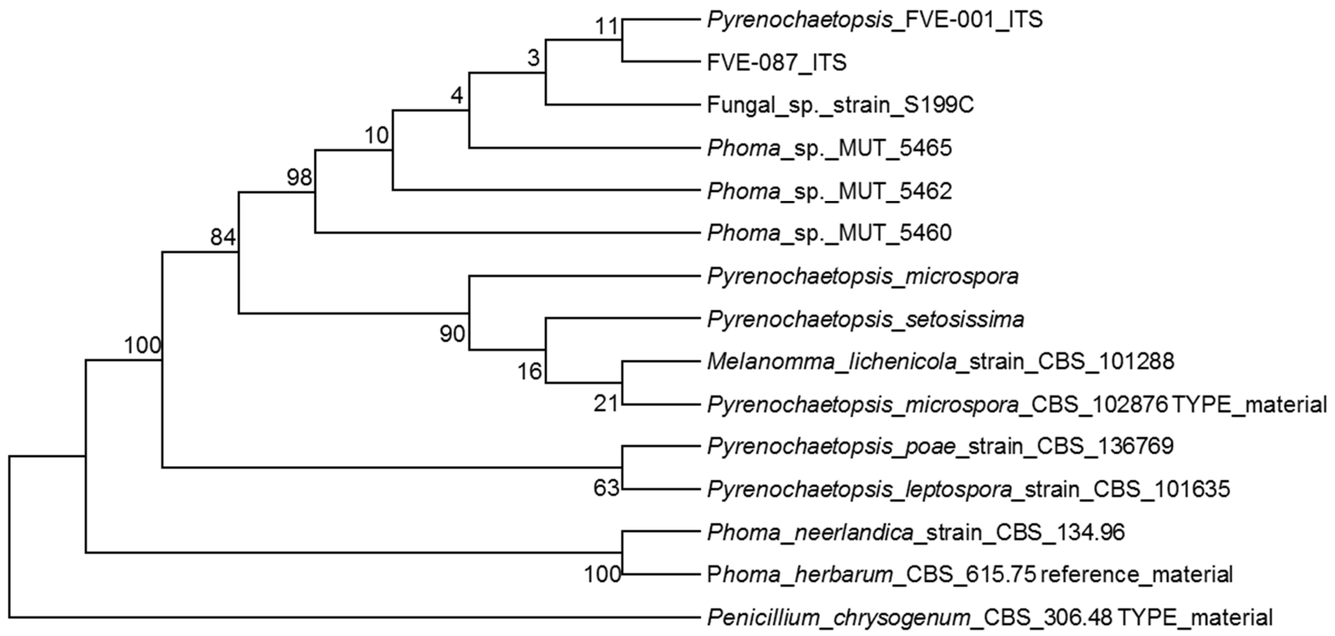

Figure S2. Structures of compounds **1**, 3'-*epi*-**1**, and 5'-*epi*-**1**.

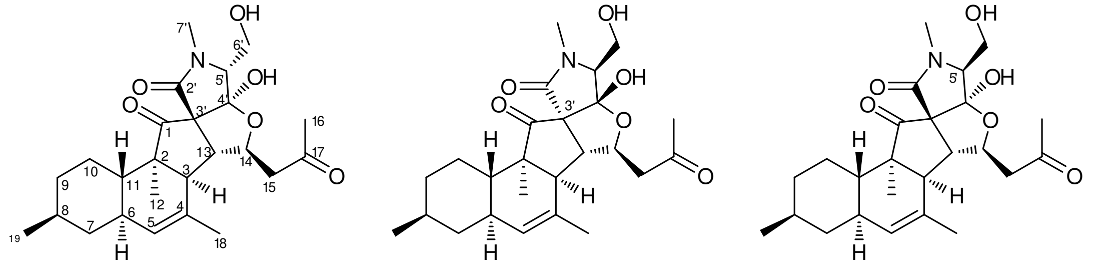

1 3'-*epi*-**1** 5'-*epi*-**1**

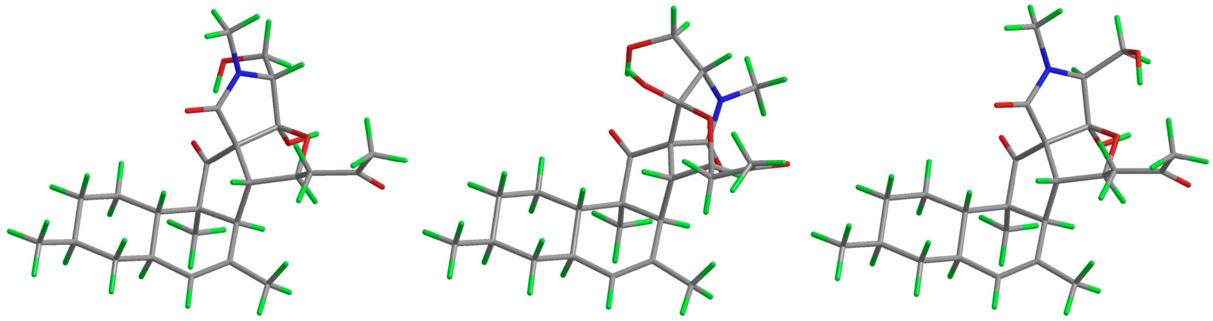

1 3'-*epi*-**1** 5'-*epi*-**1**

Figure S3. (**A**) Errors of predicted ^{13}C chemical shifts of compounds **1** and 5'-*epi*-**1**. (**B**) Errors of predicted ^{1}H chemical shifts of compounds **1** and 5'-*epi*-**1**.

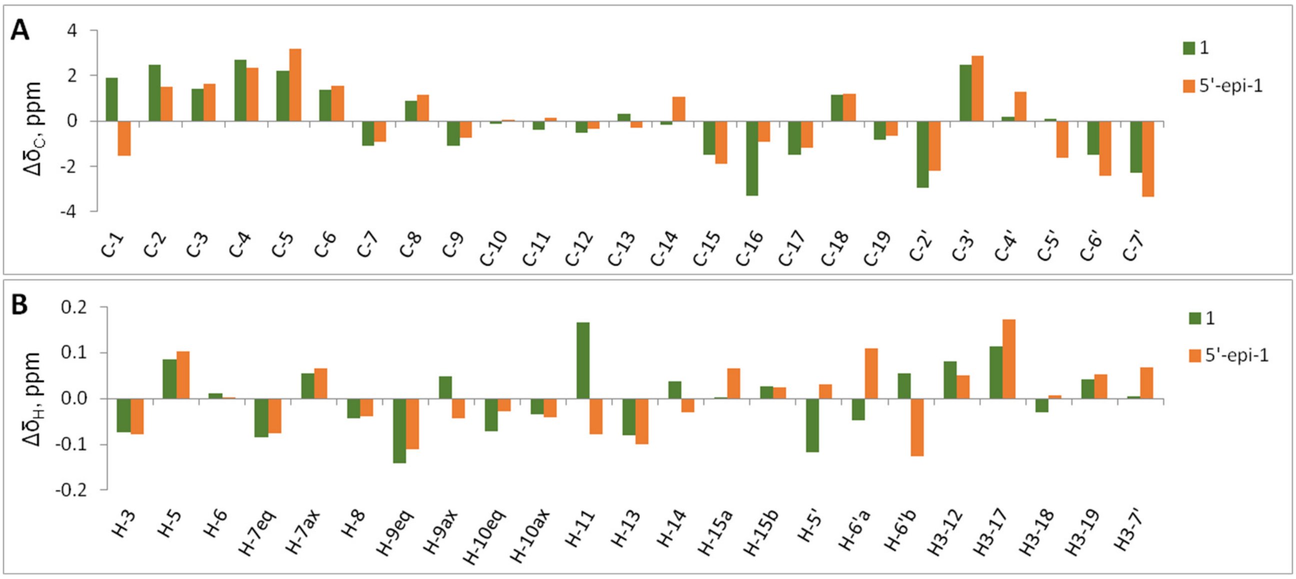

Figure S4. ^{1}H NMR spectrum of compound **1** (600 MHz, CD_3CN)

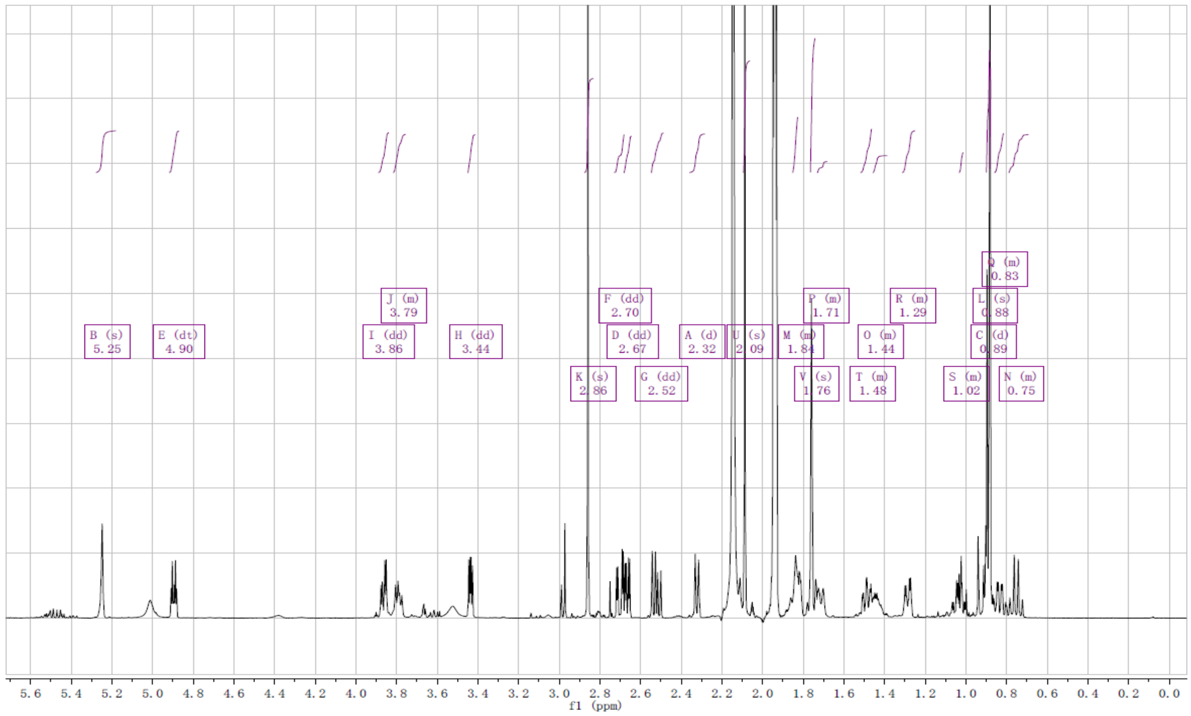

Figure S5. ^{13}C NMR spectrum of compound **1** (150 MHz, CD_3CN)

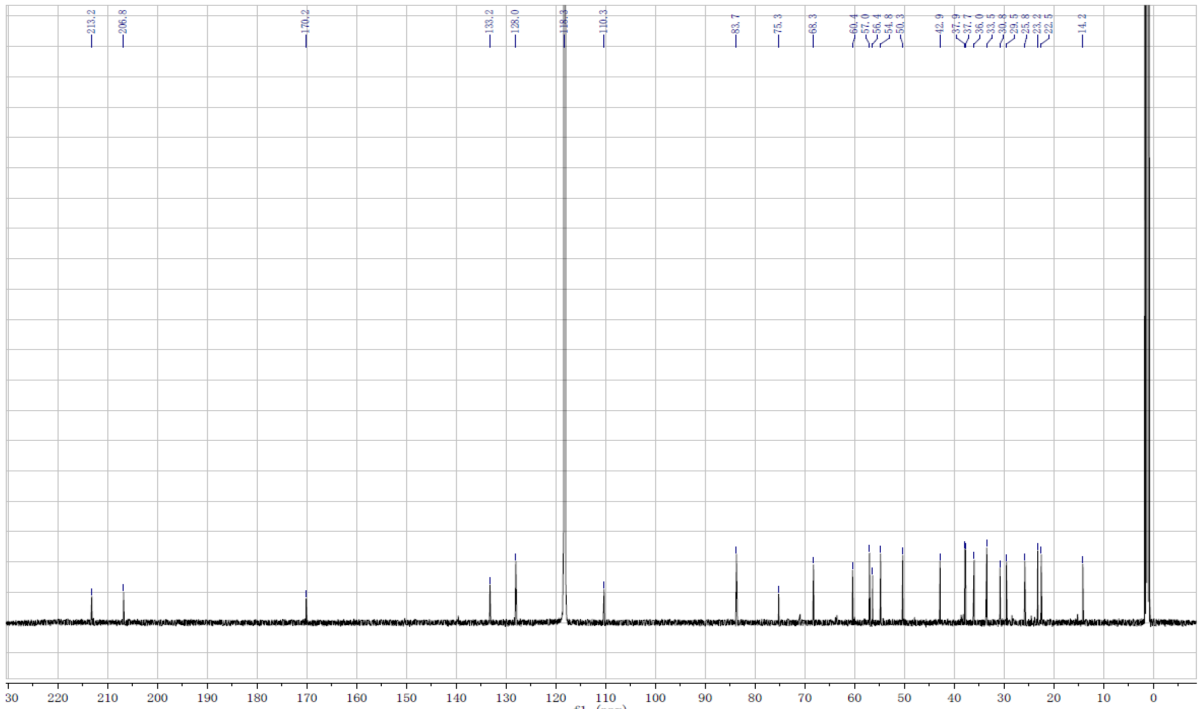

Figure S6. DEPT-HSQC spectrum of compound **1** (600 MHz, CD_3CN).

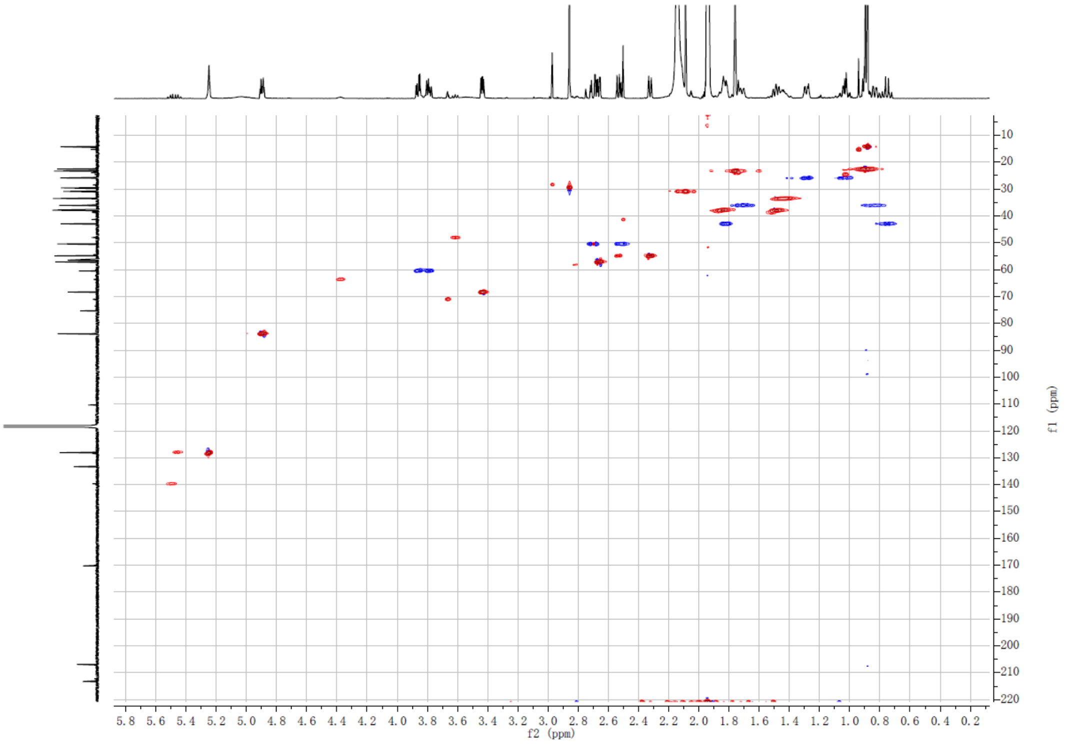

Figure S7. COSY spectrum of compound **1** (600 MHz, CD_3CN)

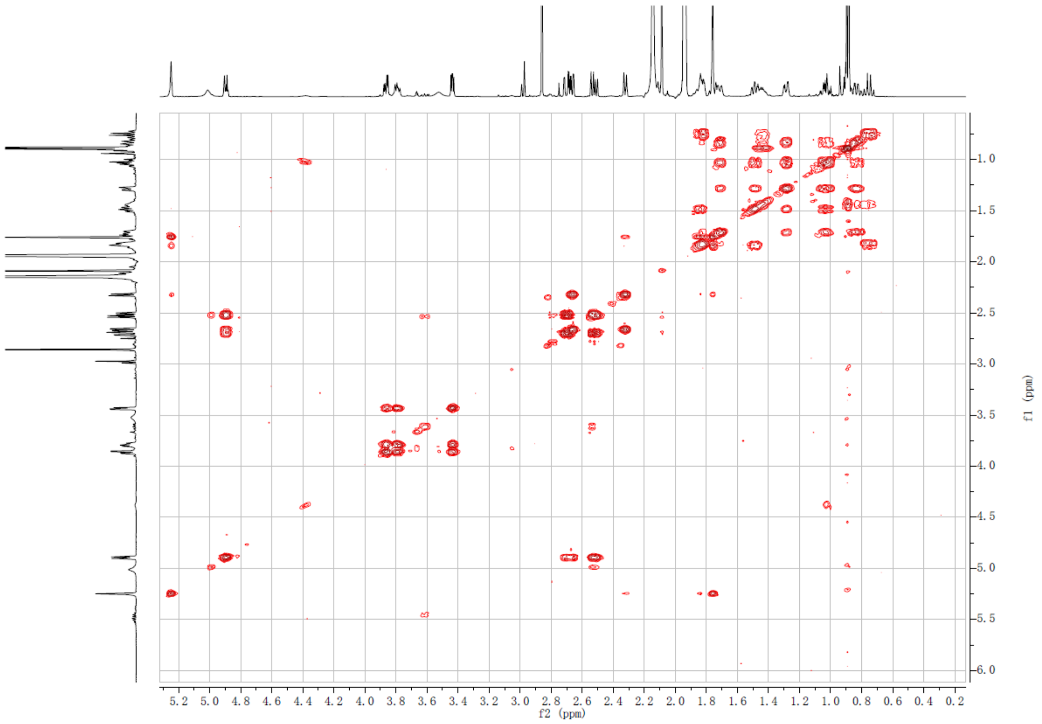

Figure S8. HMBC spectrum of compound 1 (600 MHz, CD3CN)

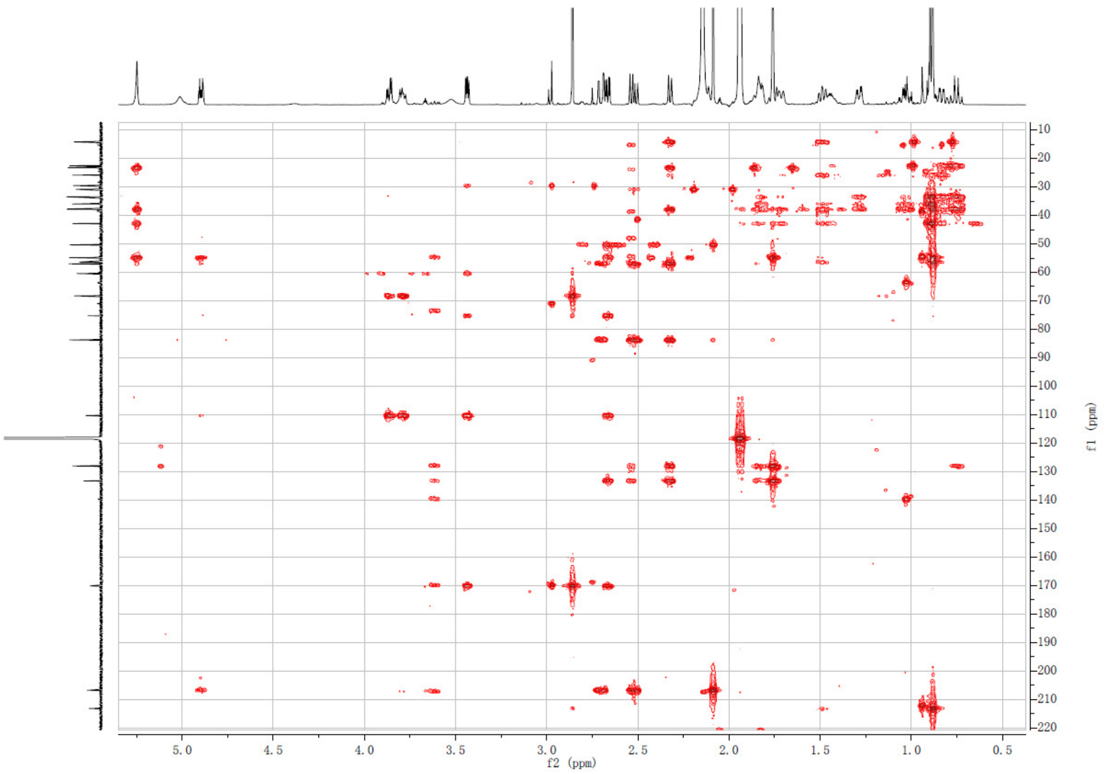

Figure S9. NOESY spectrum of compound 1 (600 MHz, CD3CN)

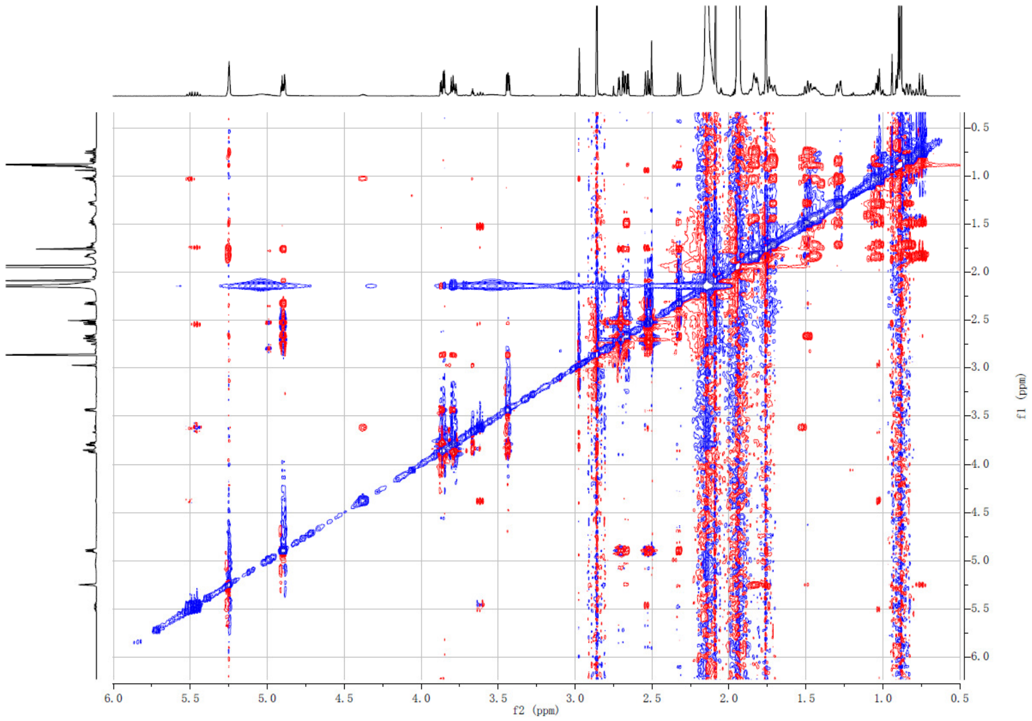

Figure S10. HR-ESIMS spectrum of compound **1**

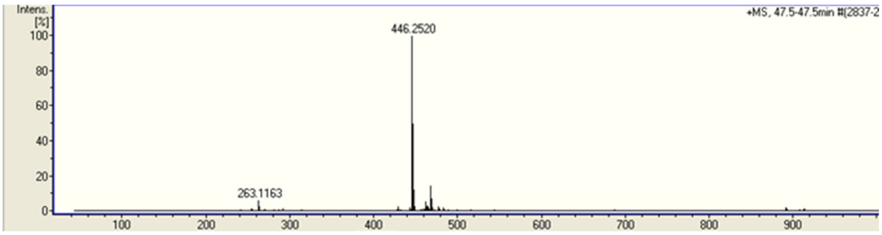

Figure S11. FT-IR spectrum of compound **1**

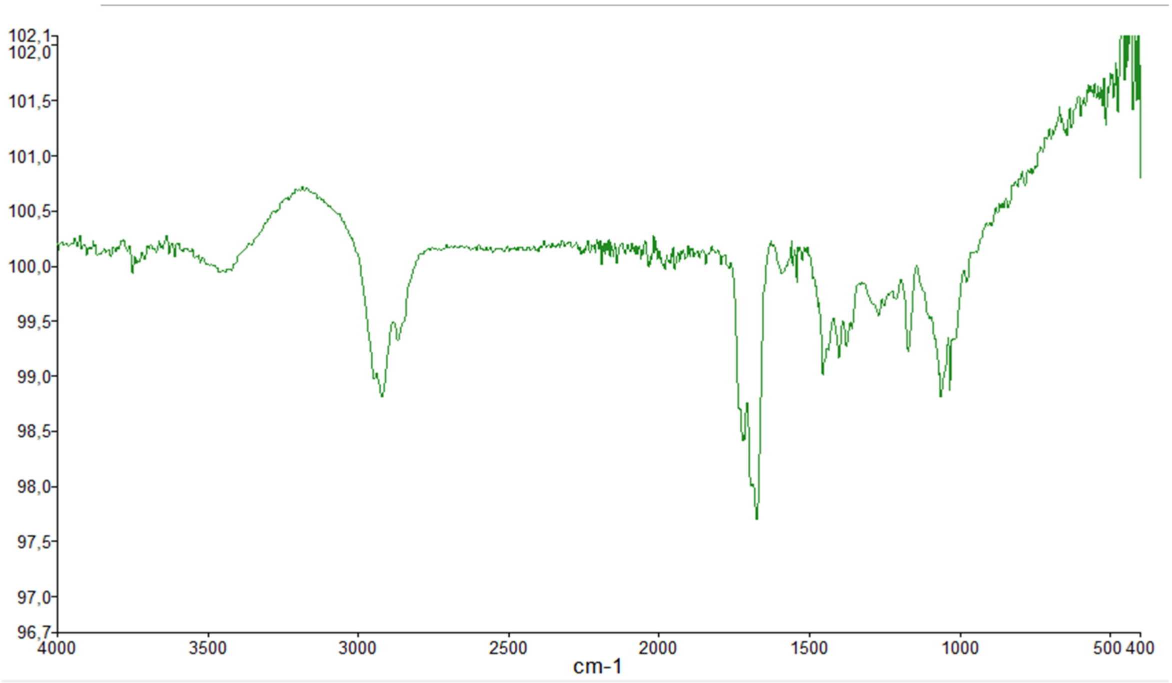

Table S1. Cartesian coordinates, energies, population at 298 K, and calculated optical rotations of significantly populated conformers of compound **1**. Conformers were optimized at the B3LYP/TZVP/SMD(ACN) level; optical rotation were calculated at the B3LYP/TZVP/SMD(MeOH) level.

Conformer 1				Conformer 2				Conformer 3			
Relative energy (kcal/mol)			0.00	Relative energy (kcal/mol)			0.65	Relative energy (kcal/mol)			0.74
Population (%)			38.7	Population (%)			13.0	Population (%)			11.1
Calcd. $[\alpha]_D$			-98.0	Calcd. $[\alpha]_D$			-152.0	Calcd. $[\alpha]_D$			-60.4
C	0.37784	-1.20880	-0.98624	C	0.37737	-1.34617	-0.78471	C	0.35542	-1.14535	-1.01927
C	1.33882	-0.08551	-1.33146	C	1.37491	-0.30065	-1.27162	C	1.33359	-0.02559	-1.32422
C	0.45876	1.18190	-1.15193	C	0.56788	1.02286	-1.17951	C	0.48067	1.24912	-1.07753
C	1.24501	2.45527	-0.93746	C	1.41977	2.26895	-1.09615	C	1.29449	2.49540	-0.81247
C	2.53958	2.42724	-0.61405	C	2.72472	2.20334	-0.82419	C	2.59215	2.42770	-0.50819
C	3.35147	1.18120	-0.41803	C	3.48534	0.93837	-0.55724	C	3.38191	1.15875	-0.38199
C	4.32704	1.29607	0.76509	C	4.51929	1.10627	0.56884	C	4.37750	1.20042	0.78932
C	5.21728	0.05475	0.90231	C	5.35369	-0.16229	0.78398	C	5.24396	-0.06322	0.85540
C	4.35852	-1.21443	0.97161	C	4.43776	-1.37302	1.00300	C	4.36028	-1.31658	0.88309
C	3.35848	-1.31982	-0.18571	C	3.38476	-1.52863	-0.10000	C	3.34058	-1.35035	-0.26141
C	2.47423	-0.06913	-0.25011	C	2.55732	-0.24590	-0.24396	C	2.48191	-0.08034	-0.25754
C	1.86943	-0.24438	-2.75773	C	1.83571	-0.61807	-2.69606	C	1.84483	-0.12901	-2.76270
C	-0.55980	0.83623	-0.01905	C	-0.43369	0.83119	0.00427	C	-0.53360	0.87107	0.04871
C	-1.98247	1.41987	-0.18158	C	-1.81931	1.49899	-0.16331	C	-1.94702	1.48727	-0.06764
C	-2.45554	2.27802	0.97645	C	-2.21757	2.42274	0.97299	C	-2.39456	2.27987	1.15445
C	-3.77482	2.99401	0.74569	C	-3.50296	3.19879	0.74446	C	-3.81767	2.80414	1.09308
C	-4.51511	3.42281	1.98304	C	-4.22431	3.65889	1.98177	C	-4.23732	3.57082	-0.13357
C	0.51531	3.75152	-1.15882	C	0.74028	3.57998	-1.38200	C	0.58697	3.81391	-0.96099
C	6.15879	0.16582	2.10008	C	6.35140	0.00623	1.92836	C	6.20706	-0.02508	2.04055
C	-0.84124	-1.41805	1.24444	C	-0.89885	-1.31350	1.40301	C	-0.85371	-1.43353	1.20845
C	-0.75917	-0.68759	-0.10454	C	-0.73838	-0.67206	0.02261	C	-0.76521	-0.64284	-0.10595
C	-2.18283	-0.87773	-0.67354	C	-2.16172	-0.79884	-0.57539	C	-2.19456	-0.78054	-0.67527
C	-2.80892	-2.07556	0.08954	C	-2.85945	-1.92895	0.21799	C	-2.84053	-1.99858	0.03727
C	-2.88131	-3.41574	-0.65426	C	-2.94460	-3.26561	-0.52439	C	-2.94570	-3.30142	-0.76670
C	-2.37211	-2.93405	2.43375	C	-2.54761	-2.68736	2.61863	C	-2.40826	-2.97090	2.33776
H	-0.12109	1.30449	-2.07339	H	-0.03405	1.09814	-2.09214	H	-0.10452	1.42561	-1.98690
H	3.07329	3.36918	-0.50804	H	3.30456	3.12376	-0.81325	H	3.14495	3.35345	-0.36446
H	3.97157	1.04415	-1.31573	H	4.05524	0.69293	-1.46507	H	3.98576	1.05097	-1.29458
H	4.95028	2.18666	0.63544	H	5.17841	1.94799	0.33302	H	5.01669	2.08356	0.69002
H	3.75698	1.44097	1.69102	H	3.99978	1.36335	1.50027	H	3.82481	1.31404	1.73000
H	5.83037	-0.00954	-0.00608	H	5.92244	-0.34017	-0.13806	H	5.84074	-0.09894	-0.06535
H	5.00535	-2.09702	0.98169	H	5.04061	-2.28398	1.06915	H	4.98849	-2.21169	0.84363
H	2.75047	-2.21773	-0.05821	H	2.74056	-2.37921	0.12962	H	2.71567	-2.24031	-0.16344
H	3.90434	-1.43933	-1.12652	H	3.88378	-1.75770	-1.04665	H	3.86959	-1.43970	-1.21506
H	1.97802	0.02137	0.72247	H	2.11040	-0.04308	0.73627	H	1.99982	-0.02327	0.72473
H	-0.13990	1.10696	0.94886	H	0.03535	1.13788	0.93867	H	-0.09989	1.08856	1.02385
H	-2.06179	1.98174	-1.11336	H	-1.87302	2.03912	-1.10999	H	-2.01649	2.10286	-0.96658
H	-2.51088	1.68464	1.89221	H	-2.27928	1.86723	1.91159	H	-2.29231	1.67150	2.05402
H	-1.71996	3.06696	1.17065	H	-1.43670	3.17899	1.11597	H	-1.73835	3.15047	1.25686
H	-3.83546	-1.80564	0.35429	H	-3.87411	-1.60745	0.47296	H	-3.85966	-1.72039	0.32126
H	3.80935	-1.21881	1.92105	H	3.93099	-1.26313	1.96969	H	3.82600	-1.35190	1.84033
H	-3.53414	-4.08251	-0.08734	H	-1.96150	-3.56818	-0.88199	H	-3.60765	-3.97995	-0.22496
H	-3.36820	-3.24042	-1.62118	H	-3.32557	-4.04035	0.14355	H	-3.43720	-3.07330	-1.72033
H	-3.06706	-0.99854	-2.39789	H	-2.88286	-1.56220	-2.20896	H	-3.09080	-0.83832	-2.39739
H	-0.95802	-3.48604	-1.11486	H	-4.70422	-3.08324	-1.39746	H	-1.02698	-3.38904	-1.24375
H	2.43069	-1.17081	-2.87490	H	0.97986	-0.64703	-3.37356	H	1.00842	-0.10117	-3.46401
H	1.04130	-0.26202	-3.46914	H	2.52603	0.14149	-3.06432	H	2.50812	0.70257	-3.00120
H	2.52197	0.58669	-3.02585	H	2.33441	-1.58561	-2.74710	H	2.38910	-1.05817	-2.92803
H	-3.83573	3.90313	2.69138	H	-3.52488	4.11319	2.68792	H	-5.18898	4.06871	0.04471
H	-4.91523	2.53372	2.48053	H	-4.65775	2.78704	2.48192	H	-4.35030	2.88327	-0.97655
H	-5.33387	4.09463	1.73058	H	-5.01626	4.36228	1.72972	H	-3.47692	4.30207	-0.41604
H	-0.31954	3.87513	-0.46380	H	-0.05128	3.79501	-0.65889	H	-0.23523	3.91801	-0.24803
H	1.18373	4.60464	-1.03296	H	1.45250	4.40608	-1.35284	H	1.27290	4.64762	-0.80350
H	0.09087	3.79356	-2.16719	H	0.26689	3.56909	-2.36911	H	0.14977	3.91289	-1.95989
H	6.81545	-0.70566	2.16988	H	7.02337	0.84994	1.74895	H	6.84595	-0.91221	2.06047
H	5.59588	0.23366	3.03606	H	6.96685	-0.88900	2.05237	H	5.66090	0.01255	2.98809
H	6.79153	1.05456	2.02724	H	5.83438	0.18793	2.87559	H	6.85743	0.85275	1.99630
H	-3.41590	-2.73956	2.68660	H	-1.84470	-2.51324	3.42958	H	-1.77118	-2.75145	3.19127
H	-2.25282	-3.99943	2.22405	H	-3.52620	-2.28894	2.89689	H	-3.44803	-2.77013	2.60175
H	-1.74127	-2.66826	3.27866	H	-2.64013	-3.76216	2.45426	H	-2.30664	-4.02813	2.08241
N	-1.98783	-2.12896	1.29210	N	-2.05679	-2.01472	1.43059	N	-2.01361	-2.12362	1.23048
O	0.47161	-2.35688	-1.36631	O	0.43925	-2.53561	-0.98741	O	0.42465	-2.27738	-1.44897

O	-2.87210	0.27548	-0.26463	O	-2.77919	0.41487	-0.20496	O	-2.85904	0.36860	-0.20966
O	-4.19404	3.23058	-0.37004	O	-3.90959	3.45683	-0.37123	O	-4.58024	2.63000	2.02411
O	0.00013	-1.34143	2.12953	O	-0.10148	-1.20589	2.32405	O	-0.00527	-1.41430	2.08956
O	-2.15440	-1.00219	-2.07051	O	-2.14733	-0.97330	-1.96157	O	-2.17621	-0.84004	-2.07525
O	-1.65110	-4.09637	-0.80252	O	-3.78253	-3.14652	-1.68401	O	-1.73110	-3.99924	-0.95714

**Conformer 4**				**Conformer 5**				**Conformer 6**			
Relative energy (kcal/mol)			0.76	Relative energy (kcal/mol)			0.92	Relative energy (kcal/mol)			0.98
Population (%)			10.8	Population (%)			8.2	Population (%)			7.5
Calcd. [α]D			-111.8	Calcd. [α]D			-134.2	Calcd. [α]D			-102.5
C	0.37220	-1.29118	-0.80190	C	0.37633	-1.34379	-0.79126	C	0.38064	-1.20497	-0.99324
C	1.33991	-0.21319	-1.25885	C	1.37416	-0.29718	-1.27527	C	1.34843	-0.08390	-1.33319
C	0.48420	1.08014	-1.16423	C	0.56767	1.02622	-1.17922	C	0.47186	1.18668	-1.15649
C	1.29087	2.35354	-1.05048	C	1.42012	2.27176	-1.09312	C	1.25791	2.45719	-0.92672
C	2.59371	2.32923	-0.76237	C	2.72497	2.20493	-0.82089	C	2.55137	2.42491	-0.59955
C	3.39466	1.08826	-0.50145	C	3.48498	0.93906	-0.55660	C	3.36094	1.17619	-0.41056
C	4.40955	1.27879	0.63831	C	4.51791	1.10351	0.57093	C	4.33542	1.28413	0.77417
C	5.28407	0.03706	0.84947	C	5.35192	-0.16581	0.78324	C	5.22144	0.03963	0.90926
C	4.40689	-1.20519	1.04853	C	4.43567	-1.37717	0.99750	C	4.35831	-1.22673	0.97454
C	3.37229	-1.38640	-0.06797	C	3.38357	-1.52918	-0.10686	C	3.36027	-1.32610	-0.18499
C	2.50487	-0.13033	-0.21097	C	2.55646	-0.24587	-0.24729	C	2.48015	-0.07246	-0.24847
C	1.83215	-0.49741	-2.68043	C	1.83496	-0.61065	-2.70054	C	1.88338	-0.24270	-2.75793
C	-0.53206	0.83492	-0.00365	C	-0.43333	0.83170	0.00460	C	-0.55815	0.84018	-0.03459
C	-1.93709	1.45110	-0.19166	C	-1.81883	1.50014	-0.16106	C	-1.97626	1.42854	-0.20252
C	-2.39233	2.34883	0.94340	C	-2.21588	2.42390	0.97570	C	-2.46537	2.24758	0.97715
C	-3.70166	3.07816	0.69693	C	-3.49994	3.20215	0.74656	C	-3.77677	2.97894	0.74901
C	-4.44859	3.52257	1.92469	C	-4.22431	3.65848	1.98344	C	-4.53642	3.36813	1.98751
C	0.57016	3.64397	-1.32889	C	0.74144	3.58367	-1.37676	C	0.52664	3.75534	-1.13056
C	6.26489	0.22773	2.00489	C	6.34818	-0.00084	1.92940	C	6.16182	0.14475	2.10848
C	-0.90867	-1.35683	1.35898	C	-0.89337	-1.31702	1.39904	C	-0.85011	-1.39406	1.23357
C	-0.77744	-0.67894	-0.00522	C	-0.73821	-0.67167	0.01993	C	-0.76168	-0.68032	-0.12467
C	-2.20779	-0.85370	-0.61255	C	-2.16347	-0.79746	-0.57348	C	-2.19354	-0.88632	-0.70641
C	-2.86520	-2.02289	0.17413	C	-2.86159	-1.92538	0.22421	C	-2.81703	-2.06006	0.08716
C	-3.05784	-3.32799	-0.60523	C	-2.96150	-3.25846	-0.51073	C	-2.88395	-3.42301	-0.61527
C	-2.45441	-2.87013	2.52651	C	-2.53205	-2.70018	2.61694	C	-2.40109	-2.86178	2.45358
H	-0.10216	1.14595	-2.08749	H	-0.03476	1.10415	-2.09133	H	-0.09767	1.31862	-2.08459
H	3.14109	3.26879	-0.73234	H	3.30524	3.12505	-0.80785	H	3.08458	3.36553	-0.48035
H	3.98252	0.87260	-1.40525	H	4.05567	0.69600	-1.46459	H	3.98140	1.04195	-1.30839
H	5.04179	2.14492	0.41809	H	5.17744	1.94578	0.33819	H	4.96162	2.17315	0.64802
H	3.87139	1.50811	1.56635	H	3.99754	1.35802	1.50259	H	3.76453	1.42814	1.69973
H	5.86690	-0.11287	-0.06870	H	5.92180	-0.34092	-0.13863	H	5.83542	-0.02454	0.00146
H	5.03862	-2.09635	1.11369	H	5.03842	-2.28838	1.06129	H	5.00206	-2.11158	0.98390
H	2.75455	-2.25990	0.14843	H	2.73907	-2.38039	0.11962	H	2.74888	-2.22222	-0.06087
H	3.88940	-1.59116	-1.01032	H	3.88325	-1.75533	-1.05388	H	3.90785	-1.44536	-1.12482
H	2.03693	0.04805	0.76395	H	2.10939	-0.04594	0.73343	H	1.98106	0.01687	0.72273
H	-0.09518	1.14829	0.94403	H	0.03613	1.13628	0.93948	H	-0.14376	1.10774	0.93644
H	-1.99734	1.99335	-1.13628	H	-1.87324	2.04058	-1.10755	H	-2.03972	2.02739	-1.11281
H	-2.45040	1.78391	1.87669	H	-2.27892	1.86784	1.91385	H	-2.53838	1.62338	1.87084
H	-1.64325	3.13225	1.10706	H	-1.43396	3.17891	1.11930	H	-1.72864	3.02559	1.20889
H	-3.85919	-1.70114	0.49795	H	-3.87331	-1.60026	0.48218	H	-3.84536	-1.77967	0.33217
H	3.88599	-1.12040	2.01016	H	3.92804	-1.27035	1.96407	H	3.80738	-1.23117	1.92296
H	-3.24122	-4.14388	0.09872	H	-1.98316	-3.56440	-0.88787	H	-3.52438	-4.07696	-0.01999
H	-3.94508	-3.22852	-1.23054	H	-3.33477	-4.02740	0.16956	H	-3.37468	-3.27884	-1.58290
H	-2.06139	-1.92684	-2.21866	H	-2.89700	-1.55459	-2.20296	H	-2.05193	-0.35024	-2.57399
H	-1.13036	-3.56895	-1.05014	H	-3.90497	-3.85524	-2.13640	H	-0.96622	-3.49256	-1.09899
H	2.48950	0.29834	-3.03154	H	2.52593	0.14946	-3.06644	H	1.05786	-0.25777	-3.47267
H	2.37688	-1.43945	-2.73456	H	2.33296	-1.57841	-2.75438	H	2.53873	0.58706	-3.02328
H	0.98631	-0.55870	-3.36824	H	0.97918	-0.63689	-3.37828	H	2.44252	-1.17045	-2.87434
H	-5.26075	4.19792	1.66061	H	-4.65897	2.78515	2.47991	H	-4.94783	2.46442	2.44804
H	-3.77078	4.00438	2.63367	H	-5.01556	4.36275	1.73165	H	-5.34836	4.05164	1.74459
H	-4.85801	2.64051	2.42711	H	-3.52646	4.11056	2.69261	H	-3.86710	3.82137	2.72286
H	1.25430	4.49273	-1.28434	H	-0.05038	3.79776	-0.65367	H	-0.30926	3.86652	-0.43436
H	0.10630	3.62799	-2.32038	H	1.45406	4.40937	-1.34570	H	1.19356	4.60743	-0.99082
H	-0.23420	3.82497	-0.61064	H	0.26852	3.57488	-2.36412	H	0.10342	3.81222	-2.13874
H	6.90944	1.09541	1.83986	H	6.96338	-0.89650	2.05152	H	5.59793	0.21228	3.04390
H	6.90919	-0.64735	2.12593	H	5.82995	0.17807	2.87650	H	6.79763	1.03154	2.03853
H	5.73344	0.38241	2.94891	H	7.02048	0.84333	1.75341	H	6.81545	-0.72909	2.17706
H	-2.63623	-3.91945	2.29265	H	-1.86004	-2.47507	3.44155	H	-3.45459	-2.68338	2.67488
H	-1.68255	-2.80573	3.28903	H	-3.53759	-2.35316	2.86464	H	-2.25313	-3.93026	2.28032
H	-3.37913	-2.43094	2.91074	H	-2.56011	-3.78114	2.46836	H	-1.79723	-2.55641	3.30487
N	-2.00520	-2.14977	1.34966	N	-2.05207	-2.01650	1.43070	N	-2.00850	-2.08193	1.29734
O	0.48061	-2.47961	-1.02570	O	0.43789	-2.53252	-0.99784	O	0.46947	-2.35182	-1.37882
O	-2.85618	0.33009	-0.25689	O	-2.77924	0.41673	-0.20180	O	-2.87086	0.29328	-0.35009
O	-4.10678	3.31339	-0.42420	O	-3.90286	3.46488	-0.36937	O	-4.17385	3.25838	-0.36490

O	-0.13103	-1.21252	2.29168	O	-0.09167	-1.21365	2.31674	O	-0.00581	-1.31619	2.11586
O	-2.26024	-0.99411	-2.00757	O	-2.15383	-0.97380	-1.95920	O	-2.29130	-1.14870	-2.07984
O	-1.99073	-3.66369	-1.49627	O	-3.88953	-3.05829	-1.59046	O	-1.64556	-4.09589	-0.74690
**Conformer 7**				**Conformer 8**				**Conformer 9**			
Relative energy (kcal/mol)			1.33	Relative energy (kcal/mol)			1.46	Relative energy (kcal/mol)			1.71
Population (%)			4.1	Population (%)			3.3	Population (%)			2.2
Calcd. [α]D			-76.9	Calcd. [α]D			-96.3	Calcd. [α]D			-59.0
C	0.34991	-1.22227	-0.87249	C	0.36284	-1.28187	-0.84506	C	0.36200	-1.15414	-1.00456
C	1.32822	-0.13394	-1.27793	C	1.36952	-0.22429	-1.28392	C	1.35317	-0.04510	-1.31565
C	0.49317	1.16400	-1.09939	C	0.58086	1.10254	-1.11377	C	0.51058	1.24033	-1.08720
C	1.32193	2.41638	-0.92508	C	1.45091	2.33103	-0.97296	C	1.32837	2.48155	-0.81260
C	2.62603	2.35551	-0.64834	C	2.75661	2.23362	-0.71443	C	2.62497	2.40668	-0.50603
C	3.40860	1.08967	-0.46158	C	3.50180	0.94646	-0.51996	C	3.40719	1.13306	-0.37872
C	4.43156	1.19827	0.68157	C	4.54526	1.03913	0.60591	C	4.40487	1.17538	0.79081
C	5.28812	-0.06677	0.81431	C	5.36445	-0.24991	0.74370	C	5.26043	-0.09487	0.86723
C	4.39336	-1.30570	0.94471	C	4.43421	-1.45896	0.90378	C	4.36538	-1.33962	0.90850
C	3.34951	-1.40515	-0.17359	C	3.37059	-1.54035	-0.19708	C	3.34581	-1.37569	-0.23594
C	2.50116	-0.12984	-0.23622	C	2.55981	-0.24083	-0.26439	C	2.49785	-0.09850	-0.24543
C	1.80487	-0.34202	-2.71749	C	1.81575	-0.46936	-2.72707	C	1.86851	-0.16846	-2.75145
C	-0.50946	0.86759	0.06139	C	-0.41108	0.85987	0.06881	C	-0.52211	0.87915	0.02664
C	-1.91073	1.50678	-0.06800	C	-1.79067	1.55068	-0.04386	C	-1.92338	1.51393	-0.10097
C	-2.33570	2.34252	1.13278	C	-2.16531	2.40233	1.16331	C	-2.39893	2.22838	1.15911
C	-3.75116	2.88689	1.07012	C	-3.56338	2.99101	1.12585	C	-3.81751	2.76410	1.09451
C	-4.17806	3.60898	-0.18070	C	-3.99101	3.72313	-0.11900	C	-4.19300	3.63117	-0.07849
C	0.62108	3.73118	-1.13001	C	0.78849	3.66405	-1.18815	C	0.62198	3.80264	-0.94363
C	6.27697	0.04095	1.97358	C	6.37340	-0.15761	1.88691	C	6.22601	-0.05351	2.05023
C	-0.88025	-1.39283	1.31019	C	-0.88918	-1.35085	1.35643	C	-0.89938	-1.38929	1.20447
C	-0.77426	-0.64137	-0.01725	C	-0.73582	-0.63852	0.01035	C	-0.77156	-0.62879	-0.12456
C	-2.21766	-0.77108	-0.60285	C	-2.16541	-0.71922	-0.57997	C	-2.19885	-0.76980	-0.73598
C	-2.87422	-1.96889	0.14026	C	-2.87349	-1.87491	0.16764	C	-2.87305	-1.94599	0.01261
C	-3.10760	-3.22886	-0.69929	C	-2.99465	-3.17055	-0.62891	C	-2.97380	-3.28277	-0.73486
C	-2.41159	-2.95945	2.42633	C	-2.53593	-2.77332	2.51625	C	-2.51940	-2.83567	2.35825
H	-0.10425	1.29031	-2.00928	H	-0.02828	1.23408	-2.01528	H	-0.05803	1.42263	-2.00744
H	3.18882	3.28327	-0.57234	H	3.34911	3.14440	-0.66089	H	3.18053	3.32931	-0.35345
H	3.98902	0.91697	-1.37929	H	4.06279	0.74264	-1.44349	H	4.00821	1.01632	-1.29195
H	5.07600	2.06615	0.50875	H	5.21368	1.88416	0.41176	H	5.05129	2.05223	0.68309
H	3.90151	1.38076	1.62448	H	4.03569	1.25067	1.55408	H	3.85442	1.30186	1.73117
H	5.86432	-0.17075	-0.11434	H	5.92336	-0.38391	-0.19168	H	5.85512	-0.14484	-0.05421
H	5.01196	-2.20833	0.95278	H	5.02529	-2.37990	0.91483	H	4.98512	-2.24095	0.87847
H	2.71979	-2.28052	-0.00430	H	2.71653	-2.39337	-0.00808	H	2.71279	-2.25889	-0.12956
H	3.85739	-1.56187	-1.13009	H	3.85891	-1.72440	-1.15901	H	3.87465	-1.47923	-1.18822
H	2.04213	-0.00194	0.75069	H	2.12318	-0.08593	0.72909	H	2.01370	-0.02853	0.73503
H	-0.05609	1.12585	1.01778	H	0.07029	1.11168	1.01311	H	-0.09334	1.09494	1.00420
H	-1.97337	2.09884	-0.98307	H	-1.83902	2.14839	-0.95654	H	-1.95776	2.19439	-0.95406
H	-2.23256	1.76242	2.05084	H	-2.06461	1.82055	2.08060	H	-2.32307	1.56204	2.01939
H	-1.66470	3.20560	1.19916	H	-1.46518	3.24320	1.21564	H	-1.73821	3.08503	1.33063
H	-3.85443	-1.64979	0.50586	H	-3.87901	-1.54922	0.44787	H	-3.89497	-1.63955	0.25261
H	3.87915	-1.27062	1.91298	H	3.93651	-1.39563	1.87928	H	3.83080	-1.35974	1.86603
H	-3.29170	-4.07549	-0.03283	H	-2.02072	-3.47557	-1.01797	H	-3.64143	-3.93513	-0.16849
H	-4.00574	-3.08224	-1.29924	H	-3.38308	-3.96308	0.01472	H	-3.44805	-3.09221	-1.70261
H	-2.12157	-1.76595	-2.26199	H	-2.91107	-1.39830	-2.23740	H	-2.00303	-0.18787	-2.58432
H	-1.19422	-3.47611	-1.19879	H	-3.94536	-3.67719	-2.28117	H	-1.05314	-3.39782	-1.19867
H	2.33529	-1.28697	-2.83040	H	2.30445	-1.43740	-2.83398	H	1.03495	-0.14193	-3.45644
H	0.95278	-0.35164	-3.40027	H	0.95445	-0.45388	-3.39809	H	2.53896	0.65526	-2.99709
H	2.47128	0.46312	-3.02762	H	2.51068	0.30243	-3.05940	H	2.40564	-1.10374	-2.90471
H	-4.31704	2.88821	-0.99144	H	-3.21091	4.40964	-0.45522	H	-3.41952	4.37622	-0.27683
H	-3.41012	4.31384	-0.50653	H	-4.91537	4.26759	0.06661	H	-5.14688	4.12126	0.10926
H	-5.11789	4.13082	-0.00829	H	-4.15789	3.00617	-0.92770	H	-4.28369	3.01452	-0.97721
H	-0.17780	3.88530	-0.39955	H	0.00292	3.85237	-0.45120	H	1.31024	4.63344	-0.78132
H	1.31868	4.56546	-1.04225	H	1.51201	4.47791	-1.11997	H	0.17795	3.91377	-1.93818
H	0.15415	3.77626	-2.11923	H	0.31149	3.71007	-2.17255	H	-0.19502	3.89900	-0.22290
H	6.93477	0.90637	1.85577	H	7.05565	0.68549	1.74802	H	6.88422	0.81790	1.99585
H	6.90750	-0.84985	2.04030	H	6.97727	-1.06651	1.95654	H	6.85688	-0.94613	2.07799
H	5.75212	0.14905	2.92770	H	5.86642	-0.02101	2.84704	H	5.68203	-0.00125	2.99832
H	-3.36026	-2.58115	2.81575	H	-2.55954	-3.84673	2.31905	H	-1.92225	-2.57311	3.22836
H	-2.53839	-4.00701	2.15031	H	-1.86572	-2.58282	3.35100	H	-3.57080	-2.63162	2.56631
H	-1.65109	-2.88709	3.19956	H	-3.54287	-2.44122	2.77757	H	-2.39987	-3.90265	2.15621
N	-1.98783	-2.16944	1.28526	N	-2.05754	-2.03446	1.36267	N	-2.08356	-2.03408	1.23243
O	0.43244	-2.39593	-1.17116	O	0.40575	-2.45875	-1.11371	O	0.42660	-2.29217	-1.41920
O	-2.84447	0.40350	-0.17810	O	-2.76413	0.48513	-0.14577	O	-2.84567	0.42447	-0.36307
O	-4.50172	2.76341	2.01893	O	-4.30001	2.89372	2.08873	O	-4.61178	2.51776	1.98162
O	-0.07500	-1.31239	2.22687	O	-0.07842	-1.30531	2.27070	O	-0.06135	-1.37474	2.09576
O	-2.29994	-0.84149	-2.00105	O	-2.16825	-0.82600	-1.97178	O	-2.28232	-0.98935	-2.11672

O	-2.06549	-3.53692	-1.62906	O	-3.91627	-2.90553	-1.70047	O	-1.75552	-3.98990	-0.87465

**Conformer 10**			
Relative energy (kcal/mol)			2.05
Population (%)			1.2
Calcd. [α]D			-140.5
C	0.38068	-1.34104	-0.80083
C	1.37588	-0.28871	-1.27685
C	0.56395	1.03100	-1.18390
C	1.41015	2.28011	-1.08607
C	2.71397	2.21764	-0.80838
C	3.47835	0.95400	-0.54624
C	4.50676	1.11947	0.58529
C	5.34438	-0.14739	0.79778
C	4.43148	-1.36214	1.00680
C	3.38408	-1.51592	-0.10176
C	2.55327	-0.23511	-0.24299
C	1.84450	-0.59799	-2.70059
C	-0.44310	0.82974	-0.00706
C	-1.82520	1.49747	-0.17543
C	-2.24553	2.39134	0.97507
C	-3.53455	3.16170	0.74606
C	-4.25403	3.62373	1.98322
C	0.72546	3.59054	-1.36192
C	6.33629	0.01878	1.94752
C	-0.89815	-1.30730	1.39884
C	-0.74083	-0.67707	0.01141
C	-2.16569	-0.80464	-0.57634
C	-2.85428	-1.94084	0.21323
C	-2.86829	-3.30386	-0.48972
C	-2.55569	-2.64682	2.63695
H	-0.03171	1.10983	-2.10036
H	3.28966	3.14044	-0.78708
H	4.05280	0.71586	-1.45309
H	5.16404	1.96456	0.35652
H	3.98226	1.37014	1.51566
H	5.91783	-0.31882	-0.12256
H	5.03693	-2.27148	1.07118
H	2.74178	-2.36973	0.12119
H	3.88808	-1.73887	-1.04720
H	2.10132	-0.03872	0.73616
H	0.02138	1.13365	0.93036
H	-1.87607	2.05541	-1.11088
H	-2.31332	1.81709	1.90191
H	-1.47133	3.14952	1.14151
H	-3.88482	-1.65146	0.43541
H	3.91994	-1.25864	1.97165
H	-1.85744	-3.56672	-0.81367
H	-3.20186	-4.05923	0.22237
H	-2.83653	-0.41480	-2.35133
H	-3.43412	-2.75082	-2.25281
H	2.53187	0.16729	-3.06233
H	2.34904	-1.56231	-2.75352
H	0.99164	-0.62894	-3.38177
H	-3.55417	4.07882	2.68832
H	-4.68745	2.75249	2.48446
H	-5.04624	4.32658	1.73061
H	-0.06756	3.79600	-0.63738
H	1.43418	4.41925	-1.32457
H	0.25325	3.58646	-2.34965
H	6.95410	-0.87504	2.06993
H	5.81437	0.19412	2.89326
H	7.00630	0.86555	1.77531
H	-2.51597	-3.73270	2.53036
H	-1.93213	-2.35354	3.47822
H	-3.58823	-2.35069	2.83130
N	-2.07184	-1.97695	1.44454
O	0.44701	-2.52764	-1.01545
O	-2.78733	0.40455	-0.25755
O	-3.94482	3.41158	-0.37007
O	-0.08968	-1.20399	2.31113
O	-2.16626	-1.01045	-1.98308
O	-3.78795	-3.34180	-1.57421

Table S2. Cartesian coordinates, energies, and population at 298 K of significantly populated conformers of compound 5'-*epi*-**1**. Conformers were optimized at the B3LYP/TZVP/SMD(ACN) level.

Conformer 1				Conformer 2				Conformer 3			
Relative energy (kcal/mol)			0.00	Relative energy (kcal/mol)			0.05	Relative energy (kcal/mol)			0.32
Population (%)			22.7	Population (%)			22.0	Population (%)			13.1
C	-0.57638	-1.28034	1.10568	C	-0.57215	-1.20718	1.13624	C	-0.56244	-1.10536	1.25696
C	-1.54097	-0.12372	1.34556	C	-1.54821	-0.05054	1.32877	C	-1.54621	0.04987	1.36668
C	-0.65721	1.12854	1.09419	C	-0.68628	1.20025	1.00447	C	-0.67292	1.27833	0.98842
C	-1.43090	2.38102	0.75084	C	-1.47824	2.41699	0.58184	C	-1.45753	2.47382	0.49787
C	-2.71846	2.33053	0.40342	C	-2.77033	2.33067	0.25950	C	-2.74208	2.37014	0.15099
C	-3.53159	1.07468	0.29540	C	-3.57068	1.06246	0.24594	C	-3.54213	1.10135	0.18235
C	-4.48502	1.09868	-0.91118	C	-4.54886	1.00609	-0.93969	C	-4.49178	0.97930	-1.02112
C	-5.37641	-0.14774	-0.97001	C	-5.42314	-0.25318	-0.91122	C	-5.36565	-0.27863	-0.94331
C	-4.52026	-1.41948	-0.92411	C	-4.54650	-1.50787	-0.81545	C	-4.49142	-1.52569	-0.76053
C	-3.54429	-1.43624	0.25808	C	-3.54892	-1.44702	0.34661	C	-3.51938	-1.40074	0.41803
C	-2.65547	-0.18708	0.24454	C	-2.67800	-0.18857	0.25006	C	-2.64992	-0.14627	0.27242
C	-2.10257	-0.17825	2.76813	C	-2.08945	-0.03924	2.76078	C	-2.11443	0.14215	2.78429
C	0.40101	0.69782	0.02779	C	0.38326	0.72861	-0.03196	C	0.40022	0.75082	-0.01803
C	1.80684	1.32449	0.17331	C	1.77842	1.38115	0.08765	C	1.81042	1.36680	0.10596
C	2.32058	2.02183	-1.07118	C	2.32417	1.92616	-1.23604	C	2.37901	1.87956	-1.22155
C	3.61355	2.79724	-0.88957	C	3.74167	2.44193	-1.08556	C	3.80671	2.36395	-1.06809
C	4.34710	3.15388	-2.15353	C	3.91632	3.90208	-0.78576	C	4.01502	3.82353	-0.79104
C	-0.69680	3.68859	0.86906	C	-0.75465	3.73590	0.58167	C	-0.73227	3.79074	0.45725
C	-6.29369	-0.12770	-2.19143	C	-6.36653	-0.31248	-2.11122	C	-6.28265	-0.40449	-2.15850
C	0.82057	-1.68712	-0.95854	C	0.84782	-1.69549	-0.89116	C	0.73218	-1.70156	-0.83447
C	0.62115	-0.79887	0.27383	C	0.62722	-0.74986	0.29200	C	0.60183	-0.72627	0.33842
C	1.98632	-0.89175	0.98008	C	1.98288	-0.78571	1.02502	C	1.98914	-0.80979	1.03237
C	2.62397	-2.20902	0.48507	C	2.69150	-2.07511	0.55019	C	2.64526	-2.10940	0.50691
C	4.14963	-2.20656	0.36214	C	4.21317	-1.97486	0.35926	C	4.15529	-2.02481	0.23061
C	2.31487	-3.50492	-1.68359	C	2.38812	-3.50528	-1.52865	C	2.19936	-3.54601	-1.53757
H	-0.10773	1.32678	2.02098	H	-0.14501	1.46451	1.91985	H	-0.13797	1.58497	1.89441
H	-3.24399	3.26172	0.20303	H	-3.30701	3.24074	0.00037	H	-3.27375	3.26621	-0.16168
H	-4.16859	1.01145	1.18948	H	-4.18798	1.04382	1.15565	H	-4.18103	1.12938	1.07685
H	-5.10766	1.99786	-0.86328	H	-5.18281	1.89854	-0.92843	H	-5.12684	1.86952	-1.07300
H	-3.89698	1.17052	-1.83452	H	-3.98073	1.03410	-1.87774	H	-3.90219	0.95725	-1.94594
H	-6.00785	-0.14118	-0.07193	H	-6.03520	-0.20675	-0.00099	H	-5.99732	-0.18362	-0.05044
H	-5.16934	-2.29942	-0.87954	H	-5.18050	-2.39372	-0.71114	H	-5.12795	-2.40502	-0.62222
H	-2.93732	-2.34236	0.21580	H	-2.92987	-2.34565	0.34231	H	-2.89759	-2.29674	0.47300
H	-4.11048	-1.47956	1.19357	H	-4.09771	-1.44703	1.29339	H	-4.08784	-1.35380	1.35188
H	-2.14064	-0.17196	-0.72320	H	-2.17767	-0.21928	-0.72518	H	-2.12708	-0.23006	-0.68714
H	0.00574	0.86388	-0.97375	H	-0.00782	0.83976	-1.04259	H	0.02849	0.85171	-1.03674
H	1.83953	2.00974	1.02054	H	1.77314	2.17333	0.83871	H	1.81934	2.16994	0.84555
H	2.42883	1.31126	-1.89486	H	2.31812	1.12830	-1.98051	H	2.35910	1.07113	-1.95439
H	1.57662	2.75436	-1.40476	H	1.66522	2.72931	-1.56897	H	1.74167	2.69296	-1.57121
H	2.36840	-2.99185	1.20732	H	2.52053	-2.84922	1.30617	H	2.51543	-2.89078	1.26536
H	-3.95222	-1.49768	-1.85927	H	-3.99535	-1.62741	-1.75632	H	-3.91983	-1.69539	-1.68116
H	4.56408	-1.94504	1.34478	H	4.64127	-1.55518	1.27834	H	4.63310	-1.60114	1.12102
H	4.48990	-3.21747	0.13481	H	4.60967	-2.98699	0.26254	H	4.53552	-3.04182	0.11878
H	2.74024	-0.81258	2.76862	H	2.69281	-0.69453	2.83144	H	1.57539	-1.55653	2.79331
H	4.26461	-0.49320	-0.51859	H	4.42150	-0.33169	-0.71330	H	4.40899	-0.37626	-0.83184
H	-2.65535	-1.10090	2.94286	H	-2.76444	0.80259	2.91812	H	-2.66944	-0.75642	3.05186
H	-1.29162	-0.13086	3.49775	H	-2.62944	-0.95748	2.98985	H	-1.30614	0.26398	3.50841
H	-2.77118	0.66176	2.95853	H	-1.26912	0.05362	3.47531	H	-2.78204	0.99842	2.88377
H	4.78094	2.24377	-2.57955	H	3.27465	4.19750	0.04852	H	5.05779	4.03194	-0.55877
H	5.14120	3.87031	-1.94972	H	3.59035	4.48367	-1.65378	H	3.37166	4.14893	0.03068
H	3.65748	3.55553	-2.89979	H	4.95618	4.13229	-0.56101	H	3.71276	4.39759	-1.67260
H	-1.35569	4.53006	0.64932	H	-0.30835	3.94063	1.56007	H	-0.32534	4.04550	1.44118
H	-0.29465	3.82174	1.87867	H	0.06243	3.75134	-0.14555	H	0.11433	3.76635	-0.23496
H	0.15465	3.74275	0.18500	H	-1.43280	4.55477	0.33670	H	-1.39755	4.59631	0.14278
H	-6.92536	0.76479	-2.19973	H	-7.01223	0.56894	-2.15330	H	-6.92764	0.47233	-2.26215
H	-6.95125	-1.00117	-2.20749	H	-7.01044	-1.19502	-2.06570	H	-6.92701	-1.28429	-2.07907
H	-5.71205	-0.13248	-3.11840	H	-5.80492	-0.35885	-3.04929	H	-5.70089	-0.50067	-3.08037
H	2.36816	-4.45790	-1.15177	H	2.49971	-4.41944	-0.94076	H	1.39154	-3.69623	-2.24958
H	1.56806	-3.57769	-2.47051	H	1.62521	-3.66082	-2.28763	H	3.12251	-3.34020	-2.08092
H	3.28707	-3.29505	-2.13204	H	3.33746	-3.27894	-2.01582	H	2.33071	-4.45582	-0.94705
N	1.92300	-2.44601	-0.77523	N	1.97067	-2.41203	-0.67403	N	1.85078	-2.43778	-0.67088
O	-0.71023	-2.41586	1.49357	O	-0.70175	-2.32840	1.56397	O	-0.63132	-2.16538	1.83686
O	2.70371	0.21430	0.46908	O	2.67565	0.34132	0.54471	O	2.68100	0.31613	0.58776
O	4.01892	3.12915	0.20672	O	4.69406	1.68978	-1.19643	O	4.74199	1.58729	-1.15949
O	0.07534	-1.71141	-1.92869	O	0.09731	-1.79464	-1.85329	O	-0.07095	-1.79799	-1.75362

O	1.85503	-0.81134	2.37267	O	1.81606	-0.71736	2.41829	O	1.97642	-0.74706	2.44358
O	4.66350	-1.36471	-0.65783	O	4.64377	-1.27383	-0.78985	O	4.52284	-1.33466	-0.94676

**Conformer 4**				**Conformer 5**				**Conformer 6**			
Relative energy (kcal/mol)			0.36	Relative energy (kcal/mol)			0.58	Relative energy (kcal/mol)			0.59
Population (%)			12.4	Population (%)			8.5	Population (%)			8.4
C	-0.57953	-1.23719	1.07885	C	-0.58549	-1.28230	1.08274	C	-0.56595	-1.15379	1.25033
C	-1.55622	-0.08567	1.30800	C	-1.55312	-0.12902	1.33582	C	-1.53098	0.01320	1.38559
C	-0.69838	1.17567	1.01220	C	-0.67439	1.12940	1.09490	C	-0.63038	1.23564	1.05151
C	-1.49018	2.39775	0.60428	C	-1.44764	2.37907	0.73972	C	-1.39429	2.46452	0.61291
C	-2.78519	2.31799	0.29259	C	-2.73598	2.32671	0.39591	C	-2.67654	2.39451	0.24887
C	-3.58795	1.05172	0.26869	C	-3.54938	1.07042	0.29617	C	-3.49506	1.13761	0.21648
C	-4.58150	1.02021	-0.90517	C	-4.51016	1.09482	-0.90476	C	-4.42828	1.07451	-1.00428
C	-5.45437	-0.24008	-0.89417	C	-5.39759	-0.15430	-0.96401	C	-5.32369	-0.17064	-0.98290
C	-4.57501	-1.49506	-0.83921	C	-4.53647	-1.42281	-0.92831	C	-4.47409	-1.43877	-0.82966
C	-3.56425	-1.45996	0.31239	C	-3.55573	-1.44099	0.24965	C	-3.51606	-1.37074	0.36517
C	-2.69526	-0.19869	0.23625	C	-2.67063	-0.18905	0.23788	C	-2.62468	-0.12686	0.27300
C	-2.08469	-0.11313	2.74505	C	-2.10959	-0.19792	2.76003	C	-2.11288	0.07365	2.79882
C	0.38158	0.72821	-0.02458	C	0.39706	0.70679	0.03857	C	0.43297	0.72007	0.02640
C	1.77032	1.38242	0.11560	C	1.79768	1.33621	0.19483	C	1.86053	1.29320	0.17890
C	2.37230	1.83062	-1.22209	C	2.34490	1.96565	-1.07180	C	2.40784	1.97935	-1.05730
C	3.75892	2.41458	-1.04446	C	3.64084	2.73861	-0.89927	C	3.71642	2.72389	-0.85587
C	3.87036	3.90586	-0.92799	C	4.41777	3.00674	-2.15880	C	4.48542	3.04831	-2.10718
C	-0.76181	3.71404	0.60140	C	-0.70864	3.68597	0.83296	C	-0.65694	3.77465	0.65797
C	-6.41335	-0.27266	-2.08281	C	-6.32130	-0.13241	-2.18055	C	-6.22305	-0.23939	-2.21579
C	0.88319	-1.65951	-0.93261	C	0.83820	-1.64998	-0.97258	C	0.67457	-1.70805	-0.88936
C	0.63127	-0.75115	0.27295	C	0.62025	-0.78788	0.27534	C	0.59169	-0.77584	0.32473
C	1.97680	-0.80752	1.05336	C	1.98287	-0.90136	1.01202	C	1.99140	-0.93024	0.97538
C	2.71279	-2.05394	0.51927	C	2.63104	-2.19131	0.47383	C	2.54810	-2.27250	0.44728
C	4.23443	-1.90774	0.35705	C	4.15785	-2.16110	0.37501	C	4.06207	-2.31069	0.22490
C	2.46749	-3.41585	-1.60749	C	2.36389	-3.42606	-1.73186	C	2.05392	-3.59142	-1.66900
H	-0.16867	1.43226	1.93838	H	-0.14010	1.33766	2.03033	H	-0.08820	1.49584	1.96744
H	-3.32047	3.23207	0.04506	H	-3.25964	3.25665	0.18533	H	-3.19461	3.31187	-0.02216
H	-4.19268	1.01612	1.18623	H	-4.17993	1.00568	1.19460	H	-4.14755	1.14261	1.10168
H	-5.21560	1.91175	-0.86601	H	-5.13536	1.99179	-0.85009	H	-5.04816	1.97637	-1.03393
H	-4.02535	1.06961	-1.84952	H	-3.92747	1.17206	-1.83106	H	-3.82538	1.07492	-1.92074
H	-6.05445	-0.21560	0.02487	H	-6.02425	-0.15405	-0.06258	H	-5.96806	-0.09505	-0.09728
H	-5.20619	-2.38441	-0.74846	H	-5.18178	-2.30557	-0.88488	H	-5.12811	-2.31040	-0.72965
H	-2.94442	-2.35730	0.28059	H	-2.94618	-2.34494	0.20181	H	-2.90981	-2.27871	0.39690
H	-4.10272	-1.48268	1.26478	H	-4.11832	-1.48955	1.18703	H	-4.09579	-1.34637	1.29293
H	-2.20342	-0.20612	-0.74392	H	-2.15858	-0.16830	-0.73137	H	-2.09187	-0.18701	-0.68253
H	-0.00439	0.85903	-1.03471	H	0.00827	0.87848	-0.96439	H	0.06749	0.86671	-0.98916
H	1.73701	2.23251	0.79895	H	1.81027	2.06991	1.00121	H	1.91657	1.96934	1.03261
H	2.43185	0.97435	-1.89601	H	2.46257	1.21522	-1.85783	H	2.51050	1.26603	-1.87935
H	1.70508	2.57654	-1.65597	H	1.61421	2.68834	-1.45359	H	1.68781	2.72970	-1.40210
H	2.55167	-2.86191	1.24072	H	2.37858	-2.99970	1.16828	H	2.32647	-3.04913	1.18914
H	-4.03489	-1.59110	-1.78914	H	-3.97226	-1.49440	-1.86632	H	-3.89270	-1.58814	-1.74765
H	4.63069	-1.48529	1.28702	H	4.54712	-1.92617	1.37261	H	4.54070	-2.04573	1.17517
H	4.65907	-2.90828	0.25605	H	4.51830	-3.15685	0.11468	H	4.36259	-3.33141	-0.01385
H	1.68912	-0.06899	2.82938	H	1.77257	-0.10926	2.77928	H	1.59566	-1.60710	2.76652
H	4.51278	-0.22883	-0.66132	H	4.28712	-0.40604	-0.41726	H	4.19052	-0.60162	-0.66159
H	-2.62703	-1.03493	2.95289	H	-2.77692	0.64060	2.96141	H	-2.68843	-0.82145	3.03288
H	-1.25720	-0.04529	3.45457	H	-2.66178	-1.12210	2.92783	H	-1.31029	0.15696	3.53466
H	-2.75349	0.72748	2.93274	H	-1.29558	-0.15833	3.48704	H	-2.76458	0.93954	2.91775
H	4.88644	4.20305	-0.67509	H	4.85789	2.06757	-2.50855	H	5.29207	3.74774	-1.89390
H	3.16898	4.28035	-0.17772	H	5.21058	3.73038	-1.97680	H	3.82197	3.45777	-2.87270
H	3.58037	4.35862	-1.88134	H	3.75629	3.36144	-2.95280	H	4.90608	2.12314	-2.51355
H	-1.44254	4.53693	0.37829	H	-0.29139	3.83110	1.83470	H	-0.27001	3.97045	1.66332
H	-0.29287	3.90926	1.57104	H	0.13258	3.72942	0.13494	H	0.20541	3.78180	-0.01444
H	0.03826	3.73295	-0.14467	H	-1.36881	4.52607	0.61210	H	-1.30853	4.60263	0.37459
H	-5.86391	-0.29703	-3.02890	H	-6.95635	0.75770	-2.18192	H	-5.62815	-0.31367	-3.13127
H	-7.06015	0.60885	-2.09632	H	-6.97563	-1.00830	-2.19680	H	-6.85184	0.65126	-2.29911
H	-7.05596	-1.15668	-2.04914	H	-5.74445	-0.13126	-3.11051	H	-6.88295	-1.11035	-2.17692
H	3.42171	-3.15869	-2.06900	H	3.34048	-3.19546	-2.15999	H	1.25053	-3.65935	-2.39835
H	2.58383	-4.34672	-1.04717	H	2.41870	-4.39296	-1.22588	H	2.99634	-3.41786	-2.19051
H	1.72087	-3.55865	-2.38487	H	1.62920	-3.48336	-2.53125	H	2.12072	-4.53406	-1.12079
N	2.01806	-2.35774	-0.72558	N	1.94997	-2.39622	-0.80030	N	1.75763	-2.50392	-0.75758
O	-0.71578	-2.37293	1.46475	O	-0.72218	-2.42333	1.45357	O	-0.63855	-2.21923	1.82046
O	2.63843	0.39028	0.71510	O	2.68825	0.24864	0.58740	O	2.71986	0.15079	0.46521
O	4.74010	1.69258	-0.98952	O	4.01500	3.13532	0.18652	O	4.10605	3.05774	0.24540
O	0.14592	-1.74381	-1.90673	O	0.10028	-1.66130	-1.94872	O	-0.12812	-1.72497	-1.81266
O	1.90509	-0.93459	2.45163	O	1.95857	-0.98615	2.41158	O	2.03576	-0.83510	2.38045
O	4.66021	-1.18338	-0.77866	O	4.67293	-1.27360	-0.60634	O	4.52597	-1.49235	-0.83793

Conformer 7				Conformer 8				Conformer 9			
Relative energy (kcal/mol)			1.04	Relative energy (kcal/mol)			1.06	Relative energy (kcal/mol)			1.12
Population (%)			3.9	Population (%)			3.8	Population (%)			3.4
C	0.53511	-1.31115	-1.01725	C	0.50115	-1.32546	-0.97172	C	0.50291	-1.32818	-0.96691
C	1.49303	-0.16839	-1.33745	C	1.47704	-0.20983	-1.33187	C	1.47711	-0.21184	-1.32970
C	0.62477	1.09597	-1.09413	C	0.63532	1.07771	-1.11286	C	0.63399	1.07509	-1.11237
C	1.41654	2.35561	-0.82661	C	1.45492	2.32759	-0.88688	C	1.45204	2.32631	-0.88826
C	2.71679	2.31048	-0.52912	C	2.75669	2.26444	-0.59923	C	2.75412	2.26527	-0.60161
C	3.52880	1.05488	-0.40969	C	3.54465	0.99645	-0.45224	C	3.54390	0.99853	-0.45369
C	4.52975	1.11539	0.75624	C	4.55525	1.06720	0.70481	C	4.55565	1.07236	0.70217
C	5.41765	-0.13312	0.82266	C	5.41869	-0.19674	0.79791	C	5.42077	-0.19034	0.79640
C	4.55485	-1.40073	0.85591	C	4.53126	-1.44563	0.86981	C	4.53492	-1.44017	0.87127
C	3.53213	-1.45333	-0.28497	C	3.49934	-1.50721	-0.26223	C	3.50199	-1.50488	-0.25965
C	2.64985	-0.19947	-0.28041	C	2.64203	-0.23627	-0.28387	C	2.64301	-0.23509	-0.28260
C	1.99677	-0.27515	-2.77848	C	1.96585	-0.36326	-2.77366	C	1.96508	-0.36725	-2.77158
C	-0.39066	0.70897	0.02923	C	-0.37412	0.73813	0.03052	C	-0.37499	0.73600	0.03155
C	-1.80188	1.33204	-0.08739	C	-1.77984	1.37562	-0.08245	C	-1.78129	1.37220	-0.08180
C	-2.26669	2.08208	1.14593	C	-2.21193	2.18348	1.12619	C	-2.21396	2.17888	1.12743
C	-3.57127	2.84115	0.98337	C	-3.50580	2.95876	0.95048	C	-3.50840	2.95325	0.95335
C	-4.27346	3.22006	2.25881	C	-4.21568	3.34863	2.21829	C	-4.21361	3.34798	2.22237
C	0.68357	3.66234	-0.95971	C	0.74708	3.64486	-1.04903	C	0.74221	3.64248	-1.05084
C	6.38381	-0.07565	2.00454	C	6.39418	-0.12814	1.97150	C	6.39747	-0.11860	1.96879
C	-0.76309	-1.63954	1.11920	C	-0.79658	-1.55893	1.19033	C	-0.80076	-1.55984	1.19258
C	-0.62343	-0.79520	-0.15157	C	-0.63633	-0.76479	-0.11206	C	-0.63643	-0.76696	-0.11034
C	-2.02128	-0.91066	-0.78946	C	-2.04526	-0.86560	-0.73296	C	-2.04381	-0.86858	-0.73499
C	-2.65187	-2.19045	-0.19941	C	-2.65998	-2.14710	-0.13428	C	-2.65541	-2.15187	-0.13781
C	-4.15728	-2.06953	0.05701	C	-4.17843	-2.09149	0.00332	C	-4.16941	-2.10749	-0.00958
C	-2.20971	-3.44307	1.97217	C	-2.29859	-3.27473	2.12328	C	-2.30598	-3.27414	2.12401
H	0.03844	1.26453	-2.00435	H	0.04208	1.23396	-2.02071	H	0.04046	1.22937	-2.02038
H	3.25331	3.24541	-0.38234	H	3.31307	3.19198	-0.48256	H	3.30930	3.19372	-0.48638
H	4.12906	0.95700	-1.32578	H	4.13596	0.86235	-1.36959	H	4.13439	0.86383	-1.37147
H	5.15370	2.00889	0.65212	H	5.19613	1.94494	0.57307	H	5.19528	1.95070	0.56833
H	3.97961	1.22243	1.69930	H	4.01432	1.20979	1.64851	H	4.01556	1.21579	1.64621
H	6.01202	-0.16136	-0.09994	H	6.00578	-0.26072	-0.12755	H	6.00692	-0.25513	-0.12959
H	5.19777	-2.28544	0.81647	H	5.15627	-2.34372	0.84904	H	5.16099	-2.33754	0.85138
H	2.92326	-2.35374	-0.18637	H	2.87346	-2.39236	-0.13590	H	2.87738	-2.39062	-0.13136
H	4.06007	-1.53300	-1.24024	H	4.01828	-1.62204	-1.21887	H	4.02017	-1.62055	-1.21660
H	2.17350	-0.14775	0.70554	H	2.17490	-0.14942	0.70406	H	2.17659	-0.14718	0.70561
H	0.04364	0.91348	1.00722	H	0.07527	0.95798	0.99836	H	0.07452	0.95686	0.99911
H	-1.86373	1.98404	-0.95952	H	-1.85002	1.99205	-0.97995	H	-1.85116	1.98964	-0.97869
H	-2.33801	1.40608	2.00177	H	-2.28036	1.54375	2.00932	H	-2.28169	1.53855	2.01024
H	-1.51579	2.83332	1.41682	H	-1.44579	2.93361	1.35412	H	-1.44828	2.92931	1.35596
H	-2.47706	-3.01254	-0.90027	H	-2.40769	-2.98310	-0.79635	H	-2.39794	-2.98567	-0.79969
H	4.02445	-1.44285	1.81519	H	4.00710	-1.45234	1.83343	H	4.01172	-1.44592	1.83542
H	-4.57520	-3.05281	0.27303	H	-4.55621	-3.00917	0.45847	H	-4.54035	-3.03345	0.43877
H	-4.34452	-1.41989	0.91399	H	-4.47692	-1.24741	0.62266	H	-4.47969	-1.26443	0.61035
H	-2.84565	-1.11596	-2.53074	H	-2.94171	-1.00234	-2.42454	H	-2.93550	-1.04364	-2.42361
H	-4.76282	-0.61865	-1.09288	H	-4.85694	-2.75293	-1.71821	H	-5.59678	-1.73742	-1.31219
H	1.15721	-0.25074	-3.47622	H	1.12074	-0.34107	-3.46477	H	2.49011	-1.31139	-2.91458
H	2.65918	0.55508	-3.02537	H	2.64153	0.44752	-3.04785	H	1.11951	-0.34690	-3.46217
H	2.54014	-1.20537	-2.94239	H	2.49030	-1.30754	-2.91791	H	2.63998	0.44355	-3.04761
H	-3.56678	3.64030	2.97840	H	-5.01207	4.06192	2.01218	H	-5.00966	4.06176	2.01665
H	-4.69051	2.31640	2.71433	H	-3.51091	3.76676	2.94117	H	-3.50609	3.76719	2.94189
H	-5.07694	3.92793	2.06182	H	-4.64239	2.45007	2.67493	H	-4.64026	2.45141	2.68299
H	-0.13676	3.74577	-0.24140	H	-0.06256	3.76541	-0.32394	H	1.43166	4.47868	-0.92432
H	1.35519	4.50715	-0.79920	H	1.43739	4.48004	-0.92049	H	0.28519	3.72120	-2.04261
H	0.23825	3.76201	-1.95493	H	0.29174	3.72526	-2.04146	H	-0.06641	3.76278	-0.32454
H	7.01912	0.81295	1.95603	H	7.03002	-1.01685	2.01058	H	7.03443	-1.00646	2.00864
H	7.03771	-0.95178	2.02474	H	5.85807	-0.06094	2.92316	H	5.86235	-0.05044	2.92094
H	5.84013	-0.04472	2.95377	H	7.04734	0.74543	1.89527	H	7.04949	0.75563	1.89036
H	-2.33479	-4.39657	1.45397	H	-3.24110	-3.02015	2.61099	H	-2.39265	-4.26160	1.66423
H	-1.39579	-3.52894	2.68813	H	-2.39662	-4.25922	1.65934	H	-1.52697	-3.30044	2.88218
H	-3.13069	-3.21181	2.51069	H	-1.51217	-3.31050	2.87340	H	-3.25510	-3.02314	2.60064
N	-1.87636	-2.40096	1.01944	N	-1.93725	-2.28472	1.12679	N	-1.94127	-2.28615	1.12686
O	0.64922	-2.46007	-1.37024	O	0.58918	-2.48536	-1.29783	O	0.59359	-2.48895	-1.28912
O	-2.70914	0.21724	-0.29672	O	-2.70514	0.26676	-0.22292	O	-2.70544	0.26301	-0.22423
O	-4.01004	3.14639	-0.10796	O	-3.92830	3.27023	-0.14542	O	-3.93543	3.26026	-0.14208
O	0.02936	-1.63765	2.05092	O	-0.00303	-1.54138	2.12079	O	-0.01030	-1.54110	2.12550
O	-1.96120	-0.91311	-2.19031	O	-2.02527	-0.86734	-2.13475	O	-2.02426	-0.87219	-2.13592
O	-4.82857	-1.58321	-1.10744	O	-4.76506	-1.89216	-1.28930	O	-4.66131	-1.97152	-1.34933

Conformer 10				Conformer 11			
Relative energy (kcal/mol)			1.62	Relative energy (kcal/mol)			1.68
Population (%)			1.5	Population (%)			1.3
C	-0.49020	-1.26136	1.00801	C	-0.49127	-1.25839	1.01136
C	-1.47724	-0.14140	1.32118	C	-1.47671	-0.13624	1.32180
C	-0.65503	1.14435	1.02912	C	-0.65297	1.14775	1.02611
C	-1.49288	2.37088	0.74874	C	-1.48956	2.37435	0.74218
C	-2.79752	2.27760	0.48387	C	-2.79425	2.28173	0.47729
C	-3.57105	0.99448	0.41278	C	-3.56924	0.99929	0.40985
C	-4.60093	0.99673	-0.72939	C	-4.59894	0.99920	-0.73252
C	-5.44867	-0.28081	-0.74696	C	-5.44808	-0.27747	-0.74637
C	-4.54533	-1.51977	-0.77593	C	-4.54614	-1.51753	-0.77128
C	-3.49514	-1.51526	0.34090	C	-3.49618	-1.51069	0.34573
C	-2.65486	-0.23365	0.29044	C	-2.65445	-0.23020	0.29137
C	-1.94826	-0.22844	2.77463	C	-1.94767	-0.21867	2.77552
C	0.34760	0.75945	-0.10541	C	0.34943	0.75852	-0.10719
C	1.74276	1.42555	-0.04744	C	1.74612	1.42179	-0.05057
C	2.15915	2.12770	-1.33401	C	2.16357	2.12265	-1.33760
C	3.56921	2.68884	-1.32934	C	3.57469	2.68094	-1.33179
C	3.97827	3.56413	-0.17377	C	3.98206	3.56067	-0.17891
C	-0.79865	3.70264	0.83062	C	-0.79400	3.70560	0.82072
C	-6.44524	-0.28044	-1.90479	C	-6.44434	-0.27967	-1.90446
C	0.82504	-1.59039	-1.13420	C	0.82258	-1.59346	-1.13109
C	0.63955	-0.72762	0.12050	C	0.63895	-0.72827	0.12248
C	2.04498	-0.76556	0.75608	C	2.04479	-0.76780	0.75767
C	2.68925	-2.06578	0.23393	C	2.68175	-2.07228	0.23781
C	4.20770	-1.98752	0.10617	C	4.19554	-2.00940	0.11445
C	2.37133	-3.32036	-1.96288	C	2.36562	-3.32711	-1.95876
H	-0.05578	1.35395	1.92220	H	-0.05368	1.35931	1.91873
H	-3.36686	3.19122	0.32706	H	-3.36254	3.19554	0.31778
H	-4.14606	0.89846	1.34513	H	-4.14453	0.90674	1.34239
H	-5.25096	1.87188	-0.62911	H	-5.24803	1.87536	-0.63505
H	-4.07732	1.10030	-1.68779	H	-4.07502	1.09926	-1.69113
H	-6.01867	-0.30841	0.19090	H	-6.01836	-0.30156	0.19142
H	-5.15719	-2.42417	-0.70332	H	-5.15902	-2.42102	-0.69594
H	-2.85905	-2.39696	0.24546	H	-2.86110	-2.39342	0.25325
H	-3.99778	-1.59248	1.30990	H	-3.99911	-1.58426	1.31487
H	-2.20215	-0.18780	-0.70697	H	-2.20165	-0.18794	-0.70617
H	-0.11504	0.91706	-1.07900	H	-0.11263	0.91456	-1.08132
H	1.79674	2.11748	0.79533	H	1.80166	2.11418	0.79183
H	2.06423	1.44741	-2.18138	H	2.06806	1.44179	-2.18444
H	1.47901	2.97080	-1.49622	H	1.48488	2.96671	-1.50060
H	2.44692	-2.86821	0.93986	H	2.43488	-2.87035	0.94615
H	-4.03652	-1.56424	-1.74672	H	-4.03719	-1.56565	-1.74183
H	4.60800	-2.92324	-0.28835	H	4.58441	-2.95460	-0.27424
H	4.49570	-1.17696	-0.56106	H	4.49587	-1.20076	-0.55433
H	2.93081	-0.80194	2.45769	H	2.93122	-0.83913	2.45673
H	4.89610	-2.53128	1.86514	H	5.61207	-1.55332	1.40181
H	-1.09603	-0.16479	3.45425	H	-2.62718	0.59981	3.01503
H	-2.62867	0.58861	3.01638	H	-2.46240	-1.15831	2.97444
H	-2.46200	-1.16919	2.97085	H	-1.09531	-0.15409	3.45489
H	4.12104	2.94862	0.71894	H	3.20423	4.29266	0.04857
H	3.19944	4.29319	0.05972	H	4.91860	4.06669	-0.40742
H	4.91283	4.07340	-0.40313	H	4.12017	2.94900	0.71719
H	-1.50106	4.52236	0.67192	H	0.00143	3.79377	0.07551
H	-0.32681	3.83948	1.80904	H	-1.49533	4.52560	0.65873
H	-0.00419	3.79399	0.08472	H	-0.32317	3.84489	1.79929
H	-7.10877	0.58739	-1.85816	H	-7.10696	0.58901	-1.86079
H	-7.06950	-1.17804	-1.89063	H	-7.06956	-1.17657	-1.88761
H	-5.92655	-0.25149	-2.86796	H	-5.92536	-0.25438	-2.86758
H	3.31315	-3.07125	-2.45471	H	2.47011	-4.28540	-1.44427
H	2.48483	-4.27708	-1.44730	H	1.59067	-3.41144	-2.71681
H	1.59313	-3.41144	-2.71686	H	3.31160	-3.08303	-2.44516
N	1.98009	-2.28713	-1.02283	N	1.97638	-2.29244	-1.01961
O	-0.56317	-2.40583	1.38743	O	-0.56595	-2.40186	1.39333
O	2.68761	0.35148	0.18711	O	2.68898	0.34625	0.18392
O	4.33045	2.45518	-2.24859	O	4.33845	2.44132	-2.24744
O	0.03779	-1.64144	-2.06847	O	0.03511	-1.64435	-2.06509
O	2.01591	-0.68717	2.15436	O	2.02069	-0.68826	2.15529
O	4.77659	-1.69856	1.39027	O	4.67596	-1.78367	1.44633

Table S3. Isotropic shieldings of significantly populated conformers of compound **1**, average isotropic shieldings over conformers, linearly scaled calculated chemical shifts, experimental chemical shifts, and errors.

Atom	Isotropic shieldings											Chemical shifts		
	1	2	3	4	5	6	7	8	9	10	Average	Calcd.	Exp.	Errors
C-1	-37.0	-32.5	-36.8	-39.2	-33.0	-36.7	-38.8	-32.7	-36.5	-31.3	-36.1	215.1	213.2	1.9
C-2	125.4	126.7	125.4	125.8	126.8	125.7	125.8	126.8	126.0	126.8	125.8	58.9	56.4	2.5
C-3	128.8	129.0	128.6	128.9	129.0	126.5	128.8	128.9	126.4	128.2	128.6	56.2	54.8	1.4
C-4	46.0	45.3	47.0	45.5	45.2	46.8	46.3	46.0	47.7	46.2	46.0	135.9	133.2	2.7
C-5	51.9	52.4	51.0	52.4	52.5	51.4	51.5	51.5	50.4	51.6	51.9	130.2	128.0	2.2
C-6	146.3	146.4	146.3	146.6	146.4	146.5	146.5	146.2	146.5	146.5	146.4	39.1	37.7	1.4
C-7	143.5	143.4	143.6	143.5	143.4	143.7	143.5	143.4	143.7	143.5	143.5	41.8	42.9	-1.1
C-8	151.2	151.2	151.1	151.3	151.2	151.3	151.2	151.0	151.1	151.2	151.2	34.4	33.5	0.9
C-9	150.7	150.6	150.7	150.7	150.6	150.8	150.7	150.5	150.8	150.7	150.7	34.9	36.0	-1.1
C-10	160.2	160.3	160.2	160.4	160.3	160.3	160.5	160.4	160.3	160.4	160.3	25.7	25.8	-0.1
C-11	148.3	147.4	148.4	147.4	147.4	148.4	147.7	147.6	148.3	147.6	148.0	37.5	37.9	-0.4
C-12	172.7	172.8	172.7	172.8	172.7	172.2	172.9	172.8	172.1	172.4	172.7	13.7	14.2	-0.5
C-13	127.1	128.0	126.2	128.6	127.9	127.8	127.6	127.0	127.0	128.3	127.4	57.3	57.0	0.3
C-14	100.7	102.0	97.4	100.1	102.1	101.2	97.1	99.2	97.8	99.3	100.3	83.5	83.7	-0.2
C-15	137.2	137.1	133.2	137.0	137.1	136.8	133.0	133.1	132.7	137.4	136.3	48.8	50.3	-1.5
C-16	-24.2	-24.1	-24.1	-23.6	-24.0	-23.9	-24.5	-24.7	-23.7	-24.3	-24.1	203.5	206.8	-3.3
C-17	155.8	155.6	159.4	155.7	155.6	155.8	159.3	159.3	159.5	155.7	156.5	29.3	30.8	-1.5
C-18	161.6	161.7	161.5	161.7	161.7	161.7	161.6	161.5	161.6	161.8	161.6	24.4	23.2	1.2
C-19	164.4	164.4	164.5	164.5	164.4	164.5	164.5	164.5	164.5	164.4	164.4	21.7	22.5	-0.8
C-2'	14.2	11.8	14.8	12.8	11.7	14.1	13.6	12.4	14.8	13.5	13.5	167.2	170.2	-3.0
C-3'	107.0	106.2	107.1	104.1	106.0	105.9	104.3	106.2	106.2	106.3	106.3	77.8	75.3	2.5
C-4'	72.7	72.6	72.5	71.1	72.6	72.3	71.0	72.3	71.7	71.7	72.3	110.5	110.3	0.2
C-5'	114.7	118.1	115.1	115.9	119.7	114.5	116.1	120.1	114.7	116.6	116.0	68.4	68.3	0.1
C-6'	126.6	123.1	126.6	126.4	123.5	127.5	126.4	123.3	127.4	124.2	125.8	58.9	60.4	-1.5
C-7'	158.6	158.6	158.6	159.1	158.5	158.7	159.1	158.5	158.7	158.5	158.7	27.2	29.5	-2.3
H-3	29.69	29.68	29.63	29.67	29.68	29.87	29.62	29.62	29.82	29.63	29.69	2.25	2.32	-0.07
H-5	26.49	26.50	26.43	26.50	26.50	26.47	26.44	26.45	26.42	26.50	26.48	5.34	5.25	0.09
H-6	30.10	30.09	30.09	30.08	30.09	30.11	30.08	30.11	30.09	30.10	30.10	1.85	1.84	0.01
H-7eq	30.22	30.23	30.19	30.24	30.23	30.22	30.19	30.19	30.19	30.23	30.22	1.74	1.82	-0.08
H-7ax	31.18	31.20	31.17	31.19	31.20	31.17	31.19	31.21	31.17	31.20	31.18	0.80	0.75	0.05
H-8	30.57	30.58	30.55	30.57	30.58	30.57	30.56	30.56	30.55	30.58	30.57	1.40	1.44	-0.04
H-9eq	30.39	30.41	30.36	30.39	30.41	30.39	30.38	30.39	30.35	30.41	30.39	1.57	1.71	-0.14
H-9ax	31.08	31.15	31.09	31.13	31.15	31.08	31.13	31.15	31.09	31.14	31.11	0.88	0.83	0.05
H-10eq	30.78	30.70	30.79	30.73	30.71	30.77	30.75	30.74	30.77	30.71	30.75	1.22	1.29	-0.07
H-10ax	30.98	31.02	30.98	31.00	31.03	30.98	31.02	31.04	30.97	31.02	31.00	0.99	1.02	-0.03
H-11	30.20	30.49	30.25	30.42	30.48	30.18	30.38	30.48	30.27	30.47	30.31	1.65	1.48	0.17
H-13	29.31	29.40	29.30	29.31	29.40	29.30	29.29	29.39	29.28	29.34	29.33	2.59	2.67	-0.08
H-14	26.89	26.91	26.94	26.82	26.91	26.88	26.87	26.97	26.91	26.75	26.89	4.94	4.90	0.04
H-15a	29.06	29.17	29.65	29.10	29.16	29.07	29.65	29.73	29.68	29.10	29.21	2.70	2.70	0.00
H-15b	29.41	29.50	29.12	29.44	29.50	29.43	29.13	29.17	29.17	29.44	29.38	2.55	2.52	0.03
H-5'	28.60	28.64	28.57	28.49	28.52	28.56	28.49	28.49	28.53	28.61	28.57	3.32	3.44	-0.12
H-6'a	28.19	28.00	28.19	27.79	27.79	28.23	27.78	27.78	28.24	27.82	28.06	3.81	3.86	-0.05
H-6'b	28.06	27.85	28.08	28.28	27.84	27.88	28.27	27.84	27.88	28.31	28.03	3.84	3.79	0.05
H_3-12 [b]	31.02	31.03	31.01	31.02	31.03	31.02	31.02	31.03	31.01	31.01	31.02	0.96	0.88	0.08
H_3-17 [b]	29.75	29.76	29.65	29.75	29.76	29.75	29.61	29.63	29.65	29.75	29.73	2.20	2.09	0.11
H_3-18 [b]	30.25	30.26	30.10	30.24	30.26	30.25	30.10	30.11	30.10	30.26	30.22	1.73	1.76	-0.03
H_3-19 [b]	31.05	31.06	31.04	31.06	31.06	31.05	31.05	31.06	31.04	31.06	31.05	0.93	0.89	0.04
H_3-7' [b]	29.03	29.06	29.04	29.06	29.05	29.02	29.06	29.06	29.03	29.05	29.04	2.87	2.86	0.01

[a] Isotropic shieldings for ^{13}C were calculated at the mPW1PW91/6-311+G(2d,p) level; isotropic shielding for ^{1}H were calculated at the WP04/aug-cc-pVDZ/PCM(ACN) level.

[b] Isotropic shieldings of methyl protons are the arithmetic mean of the shieldings of the three protons of each methyl group.

Table S4. Isotropic shieldings of significantly populated conformers of compound 5'-*epi*-**1**, average isotropic shieldings over conformers, linearly scaled calculated chemical shifts, experimental chemical shifts, and errors.

Atom	Isotropic shieldings[a]												Chemical shifts		
	1	2	3	4	5	6	7	8	9	10	11	Average	Calcd.	Exp.	Errors
C-1	-30.9	-30.6	-36.7	-30.4	-30.6	-36.9	-30.8	-31.4	-31.1	-31.2	-30.9	-32.0	211.7	213.2	-1.5
C-2	127.0	127.6	126.3	128.5	127.6	125.5	127.0	126.7	126.8	126.8	126.8	127.1	57.9	56.4	1.5
C-3	129.1	129.0	128.8	127.3	126.7	128.7	129.1	129.2	129.2	129.1	129.1	128.6	56.5	54.8	1.7
C-4	46.2	47.1	47.1	47.9	47.0	46.6	45.7	45.2	45.3	46.3	46.3	46.7	135.5	133.2	2.3
C-5	51.8	50.7	51.0	50.0	51.1	51.7	52.0	52.4	52.3	51.6	51.5	51.2	131.2	128.0	3.2
C-6	146.2	146.4	146.6	146.7	146.6	146.5	146.4	146.3	146.3	146.2	146.2	146.4	39.2	37.7	1.5
C-7	143.5	143.6	143.8	143.6	143.6	143.8	143.3	143.2	143.2	143.4	143.4	143.6	42.0	42.9	-0.9
C-8	151.2	151.1	151.1	151.1	151.3	151.1	151.2	151.2	151.1	151.0	151.0	151.2	34.7	33.5	1.2
C-9	150.4	150.5	150.8	150.5	150.5	150.8	150.4	150.4	150.4	150.3	150.3	150.5	35.3	36.0	-0.7
C-10	160.4	160.4	159.9	160.5	160.5	159.8	160.5	160.5	160.4	160.3	160.3	160.3	25.8	25.8	0.0
C-11	147.3	147.5	148.8	147.2	147.3	148.7	147.4	147.5	147.5	147.5	147.5	147.7	38.0	37.9	0.1
C-12	172.8	172.7	173.0	172.0	172.2	173.1	172.9	173.0	173.0	172.9	172.9	172.7	13.8	14.2	-0.4
C-13	129.0	127.2	127.4	128.2	129.9	129.4	128.6	128.2	128.2	127.4	127.4	128.3	56.7	57.0	-0.3
C-14	100.3	98.2	97.2	99.0	100.5	98.7	101.1	101.7	101.9	98.6	98.7	99.3	84.8	83.7	1.1
C-15	137.1	137.0	136.6	137.3	136.8	136.9	137.0	137.3	137.1	133.2	133.2	136.9	48.4	50.3	-1.9
C-16	-24.3	-28.0	-28.3	-28.6	-24.2	-23.5	-24.7	-23.9	-24.3	-24.8	-25.2	-26.1	205.9	206.8	-0.9
C-17	156.1	156.6	156.7	156.4	156.0	156.1	156.0	155.9	155.9	159.3	159.3	156.4	29.6	30.8	-1.2
C-18	161.7	161.8	161.9	161.7	161.8	161.8	161.6	161.6	161.7	161.5	161.5	161.7	24.4	23.2	1.2
C-19	164.3	164.4	164.5	164.5	164.4	164.4	164.4	164.4	164.4	164.5	164.4	164.4	21.9	22.5	-0.6
C-2'	12.9	13.4	13.9	13.5	13.0	13.4	12.7	11.7	11.6	12.5	12.3	13.1	168.0	170.2	-2.2
C-3'	106.7	106.6	104.9	106.1	105.8	104.7	106.6	106.6	106.8	106.8	107.0	106.1	78.2	75.3	2.9
C-4'	71.4	71.9	71.7	70.1	70.0	71.4	72.9	73.5	73.6	73.0	73.1	71.5	111.6	110.3	1.3
C-5'	119.0	116.7	114.5	118.3	120.9	117.1	119.8	119.3	121.7	119.4	121.6	118.0	66.7	68.3	-1.6
C-6'	127.5	128.0	127.9	127.9	126.9	127.1	121.7	123.3	123.4	123.3	123.3	127.0	58.0	60.4	-2.4
C-7'	160.3	159.9	159.7	159.6	160.1	160.2	159.9	160.0	160.0	159.9	159.9	160.0	26.1	29.5	-3.4
H-3	29.60	29.65	29.73	29.85	29.82	29.72	29.65	29.65	29.65	29.58	29.59	29.69	2.24	2.32	-0.08
H-5	26.47	26.45	26.46	26.42	26.44	26.47	26.48	26.50	26.50	26.46	26.46	26.46	5.35	5.25	0.10
H-6	30.09	30.10	30.13	30.10	30.09	30.14	30.12	30.13	30.12	30.13	30.13	30.11	1.84	1.84	0.00
H-7eq	30.22	30.21	30.20	30.21	30.21	30.20	30.21	30.21	30.21	30.20	30.21	30.21	1.74	1.82	-0.08
H-7ax	31.18	31.17	31.13	31.19	31.19	31.13	31.21	31.22	31.22	31.21	31.21	31.17	0.81	0.75	0.06
H-8	30.57	30.56	30.56	30.56	30.57	30.57	30.57	30.57	30.57	30.55	30.55	30.57	1.40	1.44	-0.04
H-9eq	30.37	30.34	30.36	30.33	30.37	30.38	30.38	30.38	30.38	30.34	30.33	30.36	1.60	1.71	-0.11
H-9ax	31.12	31.14	31.11	31.14	31.13	31.10	31.14	31.14	31.14	31.13	31.13	31.13	0.86	0.83	0.03
H-10eq	30.69	30.69	30.84	30.65	30.68	30.87	30.74	30.74	30.73	30.76	30.76	30.73	1.25	1.29	-0.04
H-10ax	31.00	30.98	30.97	30.98	30.99	30.98	31.03	31.03	31.03	31.01	31.01	30.99	0.99	1.02	-0.03
H-11	30.54	30.64	30.37	30.66	30.57	30.28	30.48	30.47	30.48	30.54	30.54	30.53	1.44	1.48	-0.04
H-13	29.35	29.30	29.30	29.30	29.35	29.33	29.39	29.40	29.40	29.35	29.35	29.33	2.59	2.67	-0.08
H-14	26.81	27.30	27.28	27.28	26.76	26.77	26.85	26.86	26.86	26.90	26.91	27.03	4.80	4.90	-0.10
H-15a	29.30	28.92	28.92	29.05	29.35	29.28	29.34	29.40	29.39	29.13	29.12	29.15	2.77	2.70	0.07
H-15b	29.02	29.87	29.84	29.88	29.04	29.01	29.03	29.08	29.08	29.67	29.67	29.44	2.49	2.52	-0.03
H-5'	28.37	28.41	28.65	28.47	28.44	28.61	28.10	28.20	28.14	28.19	28.12	28.42	3.46	3.44	0.02
H-6'a	27.92	27.95	27.87	27.85	27.82	27.85	28.06	27.84	27.83	27.85	27.83	27.90	3.97	3.86	0.11
H-6'b	28.20	28.22	28.24	28.23	28.21	28.24	28.27	28.23	28.01	28.26	28.05	28.21	3.66	3.79	-0.13
H_3-12[b]	31.06	31.05	31.05	31.06	31.05	31.07	31.06	31.06	31.06	31.04	31.04	31.06	0.93	0.88	0.05
H_3-17[b]	29.76	29.59	29.57	29.57	29.77	29.74	29.74	29.74	29.75	29.62	29.62	29.67	2.26	2.09	0.17
H_3-18[b]	30.26	30.12	30.12	30.11	30.25	30.27	30.24	30.24	30.24	30.08	30.08	30.19	1.77	1.76	0.01
H_3-19[b]	31.04	31.04	31.05	31.04	31.04	31.04	31.05	31.05	31.05	31.05	31.05	31.04	0.94	0.89	0.05
H_3-7'[b]	28.96	28.94	28.95	28.93	28.96	28.99	29.14	29.15	29.14	29.14	29.13	28.98	2.93	2.86	0.07

[a] Isotropic shieldings for ^{13}C were calculated at the mPW1PW91/6-311+G(2d,p) level; isotropic shielding for ^{1}H were calculated at the WP04/aug-cc-pVDZ/PCM(ACN) level.

[b] Isotropic shieldings of methyl protons are the arithmetic mean of the shieldings of the three protons of each methyl group.

Table S5. Selected coupling constants of compounds **1** and 5'-*epi*-**1** calculated at the B3LYP/6-31G(d,p) level and relative intensities of the corresponding HMBC peaks.

Compound **1**						
Conformer	Population	H-5'/C-2'	H-5'/C-3'	H-5'/C-4'	H-5'/C-6'	H-5'/C-7'
1	38.7%	4.8	1.7	-5.3	4.0	0.9
2	13.0%	3.7	1.6	-4.7	-2.3	1.5
3	11.1%	4.8	1.7	-5.4	4.0	0.9
4	10.8%	2.7	1.2	-5.6	4.4	1.8
5	8.2%	3.8	1.7	-4.8	-3.1	1.5
6	7.5%	5.5	1.5	-5.9	3.2	0.8
7	4.1%	2.6	1.2	-5.7	4.4	1.8
8	3.3%	3.8	1.7	-4.9	-3.2	1.5
9	2.2%	5.6	1.5	-5.9	3.2	0.8
10	1.2%	4.6	2.2	-5.1	-2.9	1.4
**Average coupling (Hz)**		**4.3**	**1.6**	**-5.3**	**2.2**	**1.2**

Compound 5'-*epi*-**1**						
Conformer	Population	H-5'/C-2'	H-5'/C-3'	H-5'/C-4'	H-5'/C-6'	H-5'/C-7'
1	22.7%	0.4	-0.2	-2.7	3.8	0.0
2	21.0%	0.8	-0.3	-2.6	3.9	0.2
3	13.1%	0.9	-0.4	-2.2	2.9	0.2
4	12.4%	0.8	-0.4	-2.8	2.8	0.2
5	8.5%	0.4	-0.3	-2.7	3.0	0.1
6	8.4%	0.5	-0.3	-2.2	3.0	0.1
7	3.9%	0.7	-0.2	-2.4	-0.5	0.4
8	3.8%	0.2	-0.1	-2.2	-2.5	0.2
9	3.4%	0.2	-0.1	-2.3	-3.0	0.2
10	1.5%	0.2	-0.1	-2.1	-2.6	0.2
11	1.3%	0.2	-0.1	-2.1	-3.1	0.2
**Average coupling (Hz)**		**0.6**	**-0.3**	**-2.5**	**2.6**	**0.2**

HMBC experiment (optimized for J_{CH} = 8 Hz)					
	H-5'/C-2'	H-5'/C-3'	H-5'/C-4'	H-5'/C-6'	H-5'/C-7'
**Relative peak intensity**	**0.822**	**0.149**	**1.000**	**0.177**	**0.072**

Table S6. Bioactivity of Kupchan subextracts (KM, KC, KH) and C18 SPE fractions (F0-F10) obtained from *Pyrenochaetopsis* FVE-087 against cancer cell lines (A-375, A-549, HT-29, HCT-116, MB-231) and HaCaT cells. The activity is expressed as % inhibition at 100 µg/mL test concentration. Reference compound: Doxorubicin.

Sample	A-375	A-54	HT-29	HCT-116	MB-231	HaCaT
**KM**	0	0	0	0	0	0
**KC**	99	99	84	99	99	98
**KH**	69	35	99	34	0	64
**F0**	0	0	0	0	0	0
**F1**	0	0	0	0	0	0
**F2**	0	0	0	0	0	0
**F3**	0	0	0	0	0	0
**F4**	0	0	0	0	23	0
**F5**	0	0	0	0	43	20
**F6**	43	0	0	0	33	45
**F7**	**98**	99	99	99	99	98
**F8**	**95**	49	74	67	68	78
**F9**	**98**	99	99	99	99	98
**F10**	27	0	0	28	66	25
**Reference**	88	73	63	90	84	91
